# *Exserohilum rostratum*: Characterization of a Cross-Kingdom Pathogen of Plants and Humans

**DOI:** 10.1371/journal.pone.0108691

**Published:** 2014-10-06

**Authors:** Kalpana Sharma, Erica M. Goss, Ellen R. Dickstein, Matthew E. Smith, Judith A. Johnson, Frederick S. Southwick, Ariena H. C. van Bruggen

**Affiliations:** 1 Department of Plant Pathology, IFAS, University of Florida, Gainesville, Florida, United States of America; 2 Emerging Pathogen Institute, University of Florida, Gainesville, Florida, United States of America; 3 Department of Pathology, Immunology, and Laboratory Medicine, College of Medicine, Division of Infectious Diseases and Global Medicine, University of Florida, Gainesville, Florida, United States of America; Charité-University Medicine Berlin, Germany

## Abstract

Pathogen host shifts represent a major source of new infectious diseases. There are several examples of cross-genus host jumps that have caused catastrophic epidemics in animal and plant species worldwide. Cross-kingdom jumps are rare, and are often associated with nosocomial infections. Here we provide an example of human-mediated cross-kingdom jumping of *Exserohilum rostratum* isolated from a patient who had received a corticosteroid injection and died of fungal meningitis in a Florida hospital in 2012. The clinical isolate of *E. rostratum* was compared with two plant pathogenic isolates of *E. rostratum* and an isolate of the closely related genus *Bipolaris* in terms of morphology, phylogeny, and pathogenicity on one C3 grass, Gulf annual rye grass (*Lolium multiflorum*), and two C4 grasses, Japanese stilt grass (*Microstegium vimineum*) and bahia grass (*Paspalum notatum*). Colony growth and color, as well as conidia shape and size were the same for the clinical and plant isolates of *E. rostratum*, while these characteristics differed slightly for the *Bipolaris* sp. isolate. The plant pathogenic and clinical isolates of *E. rostratum* were indistinguishable based on morphology and ITS and 28S rDNA sequence analysis. The clinical isolate was as pathogenic to all grass species tested as the plant pathogenic strains that were originally isolated from plant hosts. The clinical isolate induced more severe symptoms on stilt grass than on rye grass, while this was the reverse for the plant isolates of *E. rostratum*. The phylogenetic similarity between the clinical and plant-associated *E. rostratum* isolates and the ability of the clinical isolate to infect plants suggests that a plant pathogenic strain of *E. rostratum* contaminated the corticosteroid injection fluid and was able to cause systemic disease in the affected patient. This is the first proof that a clinical isolate of *E. rostratum* is also an effective plant pathogen.

## Introduction

Several pathogenic microbes are capable of infecting a variety of organisms, belonging to different species, genera, families or even kingdoms. There are many well-known cases of cross-species, -genus or -family host jumps, some of which have caused widespread and catastrophic epidemics not only in humans but also in animal and plant species worldwide [1], [2], [3], [4]. Host jumps occurring at a cross-kingdom level are very rare, but can occur when a microbe normally colonizing a species from one taxonomic kingdom has the capacity to colonize a species belonging to another kingdom under special circumstances [4], [5], [6]. Such special circumstances include situations when a host or pathogen is moved into a new habitat, when a new host is weakened by environmental stresses, or when a host is exposed to a pathogen in unusual ways [7]. Examples of cross-kingdom jumps are those of the plant pathogenic bacterium *Burkholderia cepacia* to humans, causing cystic fibrosis [8], the plant endophyte *Cryptococcus gattii* from forest trees to animals and humans [9], and fungal plant pathogens such as *Alternaria alternata*, *Aspergillus flavus*, *Fusarium oxysporum*, *Microascus cinereus* and *Rhizopus arrhizus* to humans, causing invasive fungal infections, frequently with lethal outcomes [10].

Pathogen host shifts represent a major source of new infectious diseases that are a threat to the health of humans, animals or plants in certain areas [7]. Of all human emerging infectious diseases that are of major public health concern, 73% are caused by zoonotic pathogens [5]. Some of these pathogens have been shown to cycle through ecosystems from animal- to plant hosts and back, and to multiply on and in plants [11]. Several other human and animal pathogens are considered of ‘environmental origin’ but they are actually primarily associated with plants [3], [12], for example the bacterial species *Burkholderia cepacia* [8] and *Pseudomonas aeruginosa* [13]. The infection strategies and epidemiology of these pathogens, whether considered primarily as plant, animal or human pathogens, are of particular interest from the perspectives of both the biology and evolution of cross-kingdom pathogenesis [14].

Fungal infections are relatively rare in mammals compared to bacterial and viral infections. There are about 1399 recognized species of human pathogens but only approximately 325 are fungi and about 8–12 species of fungal pathogens are associated with ‘life-threatening’ diseases [5]. The apparent resistance of mammals to fungal diseases is probably a combination of the vertebrate immune system, including both innate and adaptive mechanisms, and elevated body temperatures, which exceed the thermo-tolerance range of many fungi [15]. On the other hand, fungi are the most diverse and abundant plant disease organisms. Many plant pathogens can be opportunistic human pathogens but not as frequently as typical animal and human pathogens. These opportunistic pathogens primarily affect immune-deficient patients, for example *Aspergillus* spp. causing aspergillosis and aspergilloma, *Fusarium* spp. (primarily *F. solani*) causing fusariosis and *Coccidioides* sp., which is frequently associated with agricultural soils and causes valley fever or coccidiomycosis even in healthy persons [16].

Increases in non-zoonotic infectious diseases among domesticated animals [17], wild animals [18], domesticated plants [19], and wild plants [20] have run parallel to the recent rise in the emergence of zoonotic diseases among people. It has been argued that human actions modulate the interplay between pathogens, hosts and environment that might facilitate cross-taxa host jumps [3], [7], [17]. The contact of humans with different microbial species has increased substantially over recent decades for several reasons, including encroachment into wildlife habitat, deforestation and logging, exotic pet trade, intensive animal production, live animal transport, long-distance transport of produce, modern fruit and vegetable production practices, changes in food preparation and international travel [4], [5], [7], [21]. As a result of global trade and international travel, human populations and local environments are brought into close contact with pathogenic species to which they had never been previously exposed, consequently increasing the possibility of plant or animal pathogens developing into new human pathogens and vice-versa.

Animal and human pathogens can be transmitted to new hosts in many ways. Besides natural transmission, for example by insect vectors, “un-natural” or nosocomial transmission events are increasingly common in clinical settings and in the community at large. Bodily fluid transmission occurs mostly to hospital staff members, human or veterinary health care workers, including laboratory, research, emergency service, or cleaning personnel, as well as patients accidentally exposed to blood or body fluids contaminated with virus particles, bacteria, parasites or fungi [22], [23]. Transmission of cross-kingdom pathogens by contaminated needles and blood transfusions, rather than by their natural vectors, may ultimately affect the distribution patterns of infectious agents. Relatively little research has been done to document cross-kingdom jumps compared to the attention given to single-host pathogens or cross-species pathogens. Here, we present an example of possible cross-kingdom jumping of a fungal species isolated from a patient who died from meningitis in a Florida hospital in 2012 [24].

A nationwide outbreak of fungal meningitis was first detected in October 2012 at seemingly random locations in the USA. Since that time, the number of cases has totaled almost 751 patients with varying symptoms, resulting in 64 deaths across 20 states [25]. The main organism isolated from diseased patients was *Exserohilum rostratum* [24]. This outbreak was caused by contaminated corticosteroid injections from the New England Compounding Center (NECC), a compounding pharmacy in Framingham, Massachusetts. The United States health regulators confirmed the presence of *E. rostratum* in the NECC steroid vials used for injections to limit pain. More than 20 other species of fungi were also recovered from patient specimens and/or contaminated vials of medication, but *E. rostratum* seemed to be uniquely pathogenic in this setting. It was estimated that as many as 14,000 people may have been exposed to the contaminated medication [11]. There have also been other instances where *E. rostratum* has caused disease in humans and domesticated animals like cats, dogs, horses and cattle [26]. *Exserohilum* may infect both immunocompromised and immunocompetent hosts with variable clinical manifestations, ranging from cutaneous infections to fulminant disseminated disease. For example, this fungus has been documented in association with a corneal ulcer in the human eye [26] and as the causal agent of phaeohyphomycocis in children with leukemia [28]. So far, three species of the genus *Exserohilum* are reported to cause human diseases, namely *E. rostratum*, *E. longirostratum*, and *E. mcginnisii* [29].

Many *Exserohilum* species are plant pathogens that cause distinct leaf spots and blights. *E. rostratum* is a plant pathogen that affects a wide range of species, particularly grasses, Poaceae [30], but also other monocotyledonous plant families [31]. However, under certain circumstances *E. rostratum* can survive and thrive in human and animal hosts. Thus, *E. rostratum* seems to be a versatile pathogen that may be able to jump from plants to animals and humans [5]. However, the taxonomy of *Exserohilum* is not well defined [32], and the human, animal and plant pathogenic strains may be different. Isolates from meningitis patients have not been tested for pathogenicity on plants.

The main objective of this research was to address the question if *E. rostratum* associated with the meningitis originating from corticosteroid medication contaminated with this fungus could have originated from plants infected by *E. rostratum*. This would mean that *E. rostratum* could possibly be considered as an example of a cross-kingdom jumping fungal plant pathogen. To reach this objective, we compared the morphology, partial ribosomal RNA sequences and plant pathogenicity of one isolate of *E. rostratum* from a meningitis patient, who had received corticosteroid spinal injections and had died in a Florida hospital in 2012, with two isolates of *E. rostratum* and one isolate of *Bipolaris* sp. that originated from plants with leaf spots typical for *Exserohilum* or *Bipolaris* infections. With this research, we tested the following hypotheses:

- A clinical *Exserohilum* isolate from a deceased meningitis patient is morphologically and phylogenetically (based on ITS and 28S rDNA sequences) the same or closely related to *E. rostratum* isolated from diseased grasses.
- The clinical *Exserohilum* isolate is pathogenic to grasses and induces similar symptoms as plant pathogenic isolates of *E. rostratum* and a plant pathogenic isolate in a related genus, *Bipolaris*.

## Materials and Methods

### *Exserohilum* and *Bipolaris* isolates and culture maintenance

Four strains of *Exserohilum* or *Bipolaris* collected from human and plant sources were used in this study. *Exserohilum rostratum* C is a clinical isolate from a deceased patient obtained during an autopsy in a Florida hospital in 2012; *E. rostratum* P1 was isolated from tiger grass (*Thysanoleana maxima*) in a greenhouse at Apopka, Florida [30]; *E. rostratum* P2 was obtained from the culture collection of Dr. Lawrence Datnoff, University of Florida (originally from ATCC); and *Bipolaris* sp. was isolated from stilt grass in Missouri on 19 November 2011, and was obtained from the culture collection of Dr. Phil Harmon, University of Florida.

Thus, one clinical isolate of *Exserohilum rostratum* was used, but no human subjects or animals were used in the experiments. Use of *E. rostratum* in a plant pathology laboratory was approved by USDA-APHIS. No additional permits were required. An exemption letter was obtained from the IRB of the University of Florida, including the following statement: “You have received IRB approval to conduct the above-listed research project. Approval of this project was granted on 6/17/2014 by IRB-01. This study is approved as exempt because it poses minimal risk and is approved under the following exempt category/categories: 7. Non-Human”.

All fungi were grown on potato dextrose agar (PDA, Difco Laboratories, Detroit, MI) and incubated at 25°C under F20GE black light with a 12-h photoperiod.

### Morphological characterization

The fungi were transferred to fresh PDA plates, which were then incubated for ten days at 25°C under F20GE black light with a 12-h photoperiod. The colony growth and color, and conidia shape and size were examined using a compound microscope. Spore sizes were measured on 50 spores of all four *Exserohilum* or *Bipolaris* strains.

### DNA isolation and sequencing

Fungal isolates were grown on PDA (Difco Laboratories, Detroit, MI) at 25°C for 7 days. Mycelia were harvested and rinsed three times with sterile deionized water. Approximately 100 mg wet weight of mycelium from each isolate was added to a bead beater vial (Biospec 330TX, Bartlesville, OK) containing several 2 mm glass beads. Genomic DNA was extracted from the mycelium using the DNeasy Plant Mini Kit (Qiagen, Gaithersburg, MD). Reagent AP1 from the DNA extraction kit was added to the bead beater vial and mycelial cells were disrupted using a Biospec MiniBead Beater (model # 963) at ½ maximum speed for 2 minutes. RNAase was added to the vial and the standard extraction procedure was followed. Extracted fungal DNA was measured using a Nanodrop 2000 (ThermoScientific, Fair Lawn, NJ).

DNA from the four isolates was used for polymerase chain reaction (PCR), sequencing and phylogenetic analyses. Both the internal transcribed spacer (ITS) and the 28S subunit of ribosomal DNA (rDNA) regions were amplified with PCR using primer pairs ITS1-F (5′-CTTGGTCATTTAGAGGAAGTAA -3′) and ITS4 (5′-TCCTCCGCTTATTGATATGC -3′) and LROR (5′-ACCCGCTGAACTTAAGC-3′) and LR5 (5′-TCCT GAGGGAAACTTCG-3′), respectively [33], [34]. The PCR master mix consisted of 10x Buffer, 10 mM dNTPs, 25 mM MgCl_2_, DNA Polymerase, sterile deionized water, and 10 µM of each primer. Initial denaturation was at 95°C for 3 min, with 34 cycles of denaturation at 95°C for 30 sec., annealing at 55°C for 40 sec. (for both primer pairs), and elongation at 72°C for 1 min, with a final elongation at 72°C for 5 min (MyCycler Thermal Cycler, BioRad, Hercules, CA). The presence of PCR products was verified on a 1% agarose gel in TE buffer with SYBR Green I nucleic Acid stain (Lonza #50513, Basel, Switzerland). PCR products were cleaned using the Wizard SV Gel and PCR Clean-up System (Promega, Madison, WI). The concentration of purified PCR products was checked on the Nanodrop 2000 and submitted to the University of Florida Interdisciplinary Center for Biotechnology Research (ICBR) for DNA sequencing in both directions.

Resulting reads were assembled by isolate and locus and base calls were were visually checked against chromatograms using the software Geneious 6.16 (Biomatters Ltd., Auckland, New Zealand). The NCBI nucleotide database was queried for each consensus sequence using megablast. Relevant sequences were downloaded from GenBank for phylogenetic analysis (Table 1). Sequences were aligned using MUSCLE [35] as implemented in Geneious 6.16. After trimming to the GenBank sequences, the alignment was 487 base pairs long. A maximum likelihood tree of the ITS sequences was inferred using PhyML [36], [37], implemented in Geneious 6.16. The GTR +I+G substitution model was used with nearest neighbor interchange (NNI) topology search. Branch support was evaluated using 1000 bootstrap samples. The tree was rooted between the *Exserohilum* and *Bipolaris* clades.

**Table 1:** *Exserohilum* and *Bipolaris* ITS sequences used for phylogenetic analysis.

Species	Isolate	Host	GenBank Accession
*E. rostratum*	C^1^	human	KJ830936
	P1^1^	tiger grass	KJ830935
	P2^1^		KJ830934
	D087	banana	GQ478868
	Ex 1	sugarcane	KC198082
	IP 1129.80	human	HE664033
	UTHSC 09-131	human	HE664062
	ITF0706-1	pineapple	JN711431
	ITF0706-2	pineapple	JN711432
*E. mcginnisii*	ATCC 60408		AF081453
*E. longirostratum*	7728		AF163064
*E. gedarefense*	8307		AF163068
*E. neoregeliae*	IM201-E		AB607957
*E. minor*	81100b		AF163065
*E. turcicum*	ATCC 64835		KF278460
	94/1823		AF163067
*E. fusiforme*	KUC5012		GQ241279
*E. pedicellatum*	EEB 1336		AF229478
*Bipolaris*	117^1^	stilt grass	KJ830933
*B. sorokiniana*	ICMP 6233		JX256418
*B. oryzae*	MFLUCC 10-0716		JX256415
*B. cynodontis*	ICMP 6128		JX256412

1 Isolates from this study.

### Plant material and growing conditions

One C3 grass, Gulf annual rye grass (*Lolium multiflorum* Lam.), and two C4 grasses, Japanese stilt grass (*Microstegium vimineum* (Trin.) Camus) and bahia grass (*Paspalum notatum* Flugge), all belonging to the Poaceae family, were selected for this study. Gulf annual rye grass, which is used for lawn, pasture and forage production around the world, was purchased from Hancock Farm & Seed Company, Dade City, Florida. Seeds of Japanese stilt grass also known as Nepalese brown top and Asian stilt grass, were collected from a riparian habitat next to Trout Brook near Orange, Connecticut, and kindly provided by Dr. Luke Flory, Department of Agronomy, University of Florida. Bahia grass is a major pasture forage grass in Florida and throughout the southern Gulf Coast region of the United States, particularly for beef cattle production [38]. The Bahia grass for this study was purchased from Alachua County Feed and Seed store, Gainesville, Florida. Tiger grass is a C4 grass from SE Asia that is grown as an ornamental. This grass served as a source of *E. rostratum*, but was not selected for the pathogenicity tests. Most sources of tiger grass are already heavily infected by the pathogen, so that it would have been difficult to raise disease free plants as controls.

Seeds were sown into 6.4-cm square containers containing soilless Fafard 4P complete potting mix. The potting mix consisted of 45% Canadian sphagnum peat moss, 30% processed pine bark, 15% vermiculite, 10% perlite, starter nutrients, wetting agent and dolomitic limestone (Conrad Fafard, Inc., Agawam, MA). Since seeds of bahia grass germinate very slowly, bahia grass seeds were sown 20 days earlier than the seeds of rye grass and stilt grass. The numbers of plants per pot varied and were as follows: stilt grass 5–6, bahia grass 3–12 and rye grass 10–14. The plants were kept in a growth chamber with a day/night temperature of 25°C, ambient relative humidity (about 70%), and a light intensity of 8000 lux. The plants were watered daily with de-ionized water, and fertilized with Peters NPK 20-20-20 (2.1% NO_3_-N, 17.9% urea-N, 20% P_2_O_5_, 20% K_2_O; J.R. Peters Inc., Allentown, PA, USA) once every three weeks.

### Inoculum preparation, inoculation procedure and disease assessment

Pathogen cultures were maintained on V8-juice agar plates (330 ml V8 juice (Campbell Soup Company, Camden, NJ), 670 ml de-ionized water, 3 g calcium carbonate, and 15 g agar) and incubated at 25°C under F20GE black light on a 12-h photoperiod to enhance sporulation. Ten day-old cultures were used for inoculation. Inoculum was prepared by washing the conidia from cultures in petri plates using sterile de-ionized water with a surfactant, 0.01% Tween 20, and a sterile cell scraper. The conidial concentration was determined with a haemocytometer and adjusted to 1×10^4^ conidia ml^−1^. Four isolates of *Exserohilum* were used for inoculation of grass seedlings in two separate experiments: *E. rostratum* C, *E. rostratum* P2, and *Bipolaris* sp. in the first experiment, and *E. rostratum* C and *E. rostratum* P1 in the second experiment.

In the first experiment, rye grass, stilt grass and bahia grass plants were inoculated when they were approximately 12 cm tall. Inoculation was done by spraying 2.3 ml of spore suspension (1×10^4^ conidia ml^−1^) onto each pot using an atomizer sprayer (Preval Power Unit, Coal City, IL). The control consisted of a similar spray of sterile deionized water and Tween 20 (0.01%). Separate sprayers were used for each strain and the control to avoid cross contamination. Inoculation was done in a bio-safety cabinet in a BSL2 lab. After inoculation, plants were kept individually in plastic bags for 24 hours with moist paper towels to maintain high humidity. Plants were then removed from the plastic bags and placed in plastic crisper boxes with lids and kept in an air-conditioned growth chamber at 25°C, 12 h photoperiod and with a relative humidity of about 70 % and a light intensity of 8000 lux. The experiment was arranged in a randomized complete block design, and was repeated. In the first trial of this experiment, there were 5 blocks (two pots per block per treatment) and in the second trial, there were 4 blocks (three pots per block per treatment).

Plants were checked daily for six days for symptom development. Pots with the infected plants were counted to determine the number of pots with symptoms six days after inoculation. In a repeat of the first experiment (second trial), percent infection per plant per pot was estimated based on visual observations. After the final assessments, leaf tissues containing lesions were disinfected with a 10% bleach solution and washed in de-ionized water. Symptomatic tissue was aseptically removed and transferred to acidified PDA (Difco Laboratories, Detroit, MI) plates. Plates were incubated for ten days at 25°C, and colony growth and color, and conidia shape and size were examined using a compound microscope.

In the second experiment, Gulf annual rye grass and Japanese stilt grass were tested for their reaction to *E. rostratum* C and *E. rostratum* P1. Growing conditions of plants, inoculum preparation, inoculation and disease assessment were done according to the methods described for the first experiment, except that there were 4 blocks (four pots per block per treatment) and the numbers of plants per pot were five for each species. The experiment was arranged in a randomized complete block design, and was repeated. Plants were checked daily for five days for symptom development. Percent infection per plant per pot was estimated based on visual observations. After final disease assessment, symptomatic tissue was aseptically removed and transferred to acidified PDA and colony growth and color, and conidia shape and size were examined as described above.

### Statistical analysis

Counts of pots with symptoms after inoculation with the different *Exserohilum* and *Bipolaris* isolates were compared using a Chi^2^ test in Microsoft Excel. Average disease intensity per plant (%) was calculated per block for the second trial. Analysis of variance (PROC GLM, SAS software version 9.2; SAS Institute Inc., Cary, NC) was used to determine significant differences in disease intensity (%) values among the *Exserohilum* and *Bipolaris* isolates, plant species and their interaction. Means for isolates and plant species were separated using Tukey's test. In the second experiment, areas under disease progress curve (AUDPC) values were calculated and compared with a generalized model analysis of variance using SAS statistical software (PROC GLM, SAS software version 9.2; SAS Institute Inc.). There were no effects of repetition or repetition–treatment interactions; therefore, the data of the repeated experiments were analyzed together. Furthermore, these data were log-transformed and regressed on time without intercept to quantify the relationship between log (% infection) and days after inoculation for individual combinations of C and P isolates of *E. rostratum*, rye grass, stilt grass, and blocks. Estimates for the slope were compared with a generalized model analysis of variance. Means were separated using Tukey's test.

## Results

### Morphological characterization

**Figure 1:**
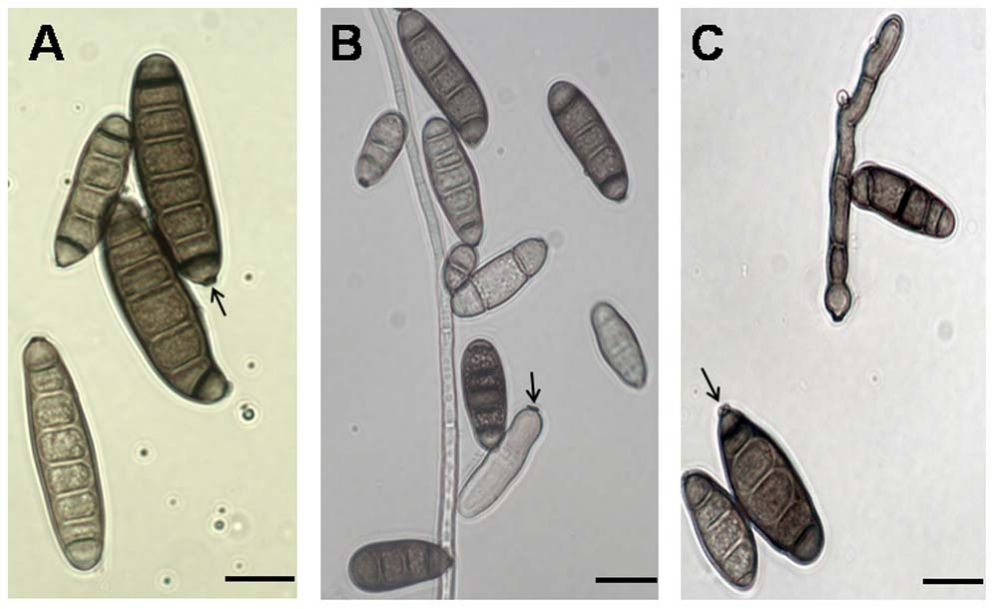
Conidia of (A) clinical isolate of *E. rostratum*, (B) *Bipolaris* sp. (C) *E. rostratum* from plant (P1) with hilum (marked by arrow) viewed under the compound microscope (bars  = 10 µm).

The colony characteristics of *Exserohilum* and *Bipolaris* strains on acid PDA plates were the same. The colonies were circular, deep-brown on the upperside and black on the underside with abundant aerial mycelium that appeared woolly or cottony. Conidia of *E. rostratum* C and P1 were indistinguishable in shape and size (P = 0.9853). Conidia of both strains were straight, curved or slightly bent, ellipsoidal to fusiform, and septate (typically 4–8 septa per conidium), 20.0–65.5 µm×11.0–20.0 µm (average 30.0×16.0 µm) (Fig. 1A). Conidia had a strongly protruding, truncate hilum and the septum above the hilum was usually thickened and darker than that between cells. Conidia had a slightly protruding and truncate hilum. Conidia of *E. rostratum* P2 were fusoid, obclavate, straight, or curved, septate (typically 3–8 septa per conidium), 15.0–44.0 µm×13.0–18.0 µm (average 18×13 µm) (Fig. 1C). The hilum was strongly protruding. Conidia of *Bipolaris* sp. were cylindrical, rounded at both ends, small, brown, smooth septate (typically 2–7 septa per conidium), 8.0–33.0 µm×6.4–10.0 µm (average 22.0×9.2 µm) (Fig. 1B).

### Phylogenetic analysis

**Figure 2:**
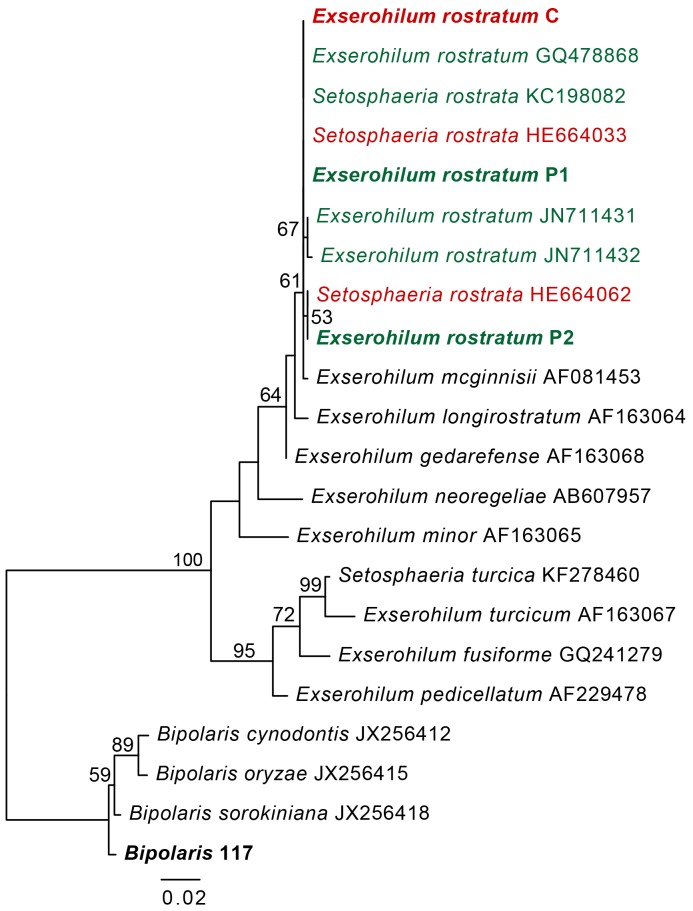
Maximum likelihood phylogeny of the *Exserohilum* and *Bipolaris* isolates relative to other *E. rostratum* clinical and plant isolates and *Exserohilum* species. Isolates used in this study are shown in bold. *E. rostratum* isolates from plants are shown in green and clinical isolates are shown in red. Bootstrap values are shown for branches with values greater than 50%. Branch lengths are in substitutions per site.

The sequences for the conserved 28S ribosomal RNA gene were identical at all sequenced nucleotides among the three *E. rostratum* isolates and differed from the *Bipolaris* isolate by three nucleotides. BLAST analysis produced a match to a single 28S sequence from an *Exserohilum* isolate (the teliomorph *Setosphaeria monoceras*, accession AY016368), which was 100% identical to the three *E. rostratum* isolates. Likewise, the *Bipolaris* isolate had 100% identical 28S sequence to a number of different *Bipolaris* (teleomorph: *Cochliobolus*) species. The ITS region contains more variation than 28S and can be used to distinguish among *Bipolaris* species [39]. *Exserohilum rostratum* C and P1 had 100% identical ITS sequences (Fig. 2). There was a single nucleotide difference between *E. rostratum* P2 ITS and the other *E. rostratum* isolates. The *E. rostratum* isolates had identical ITS sequences to other *E. rostratum* isolates from plant and human infections (Fig. 2). The ITS sequence from the *Bipolaris* isolate was 100% identical to four *B. sorokiniana* ITS sequences in GenBank and exhibited 98.2% identity to the ITS sequence of the *B. sorokiniana* type strain (accession JX256418).

### Pathogenicity tests

**Figure 3:**
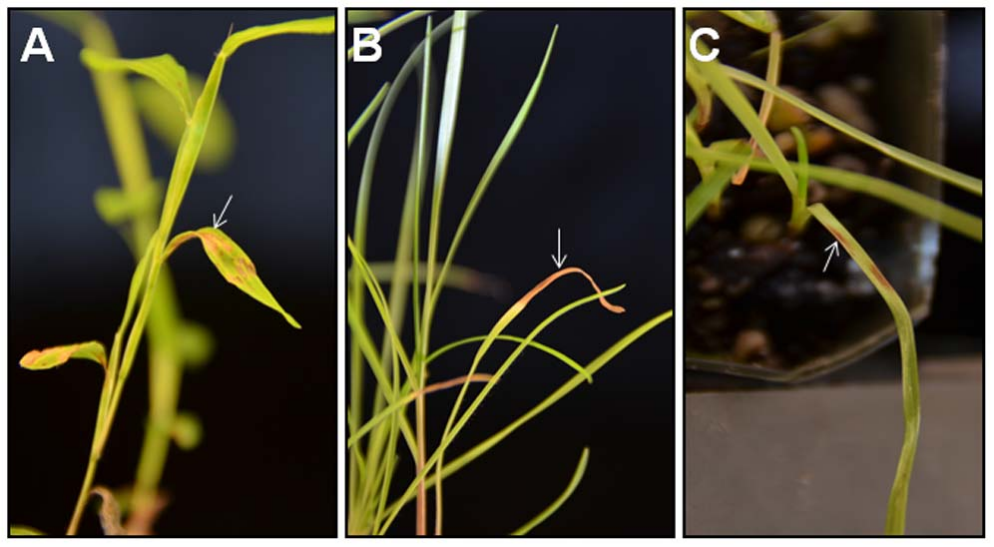
Exserohilum leaf spot (marked by arrow) on (A) stilt grass, (B) bahia grass and (C) rye grass after inoculation with the clinical isolate of *Exserohilum rostratum* at six days after inoculation.

In both trials of the first experiment, symptoms developed on all plant species two days after inoculation with all strains of *Exserohilum* or *Bipolaris*. The disease frequently occurred on leaf margins and first appeared as small water-soaked lesions. Initially the lesions on the leaf were small, elliptical and gray-green in color. As the disease progressed, the lesions expanded and became tan in color. They were not restricted by the leaf veins (Fig. 3). Eventually, the lesions coalesced and covered most of the leaf by seven days after inoculation. The lesions produced dark gray spores on the lower leaf surface giving them a dusty appearance under moist conditions. No symptoms were observed on control plants throughout the whole assessment period.

**Figure 4:**
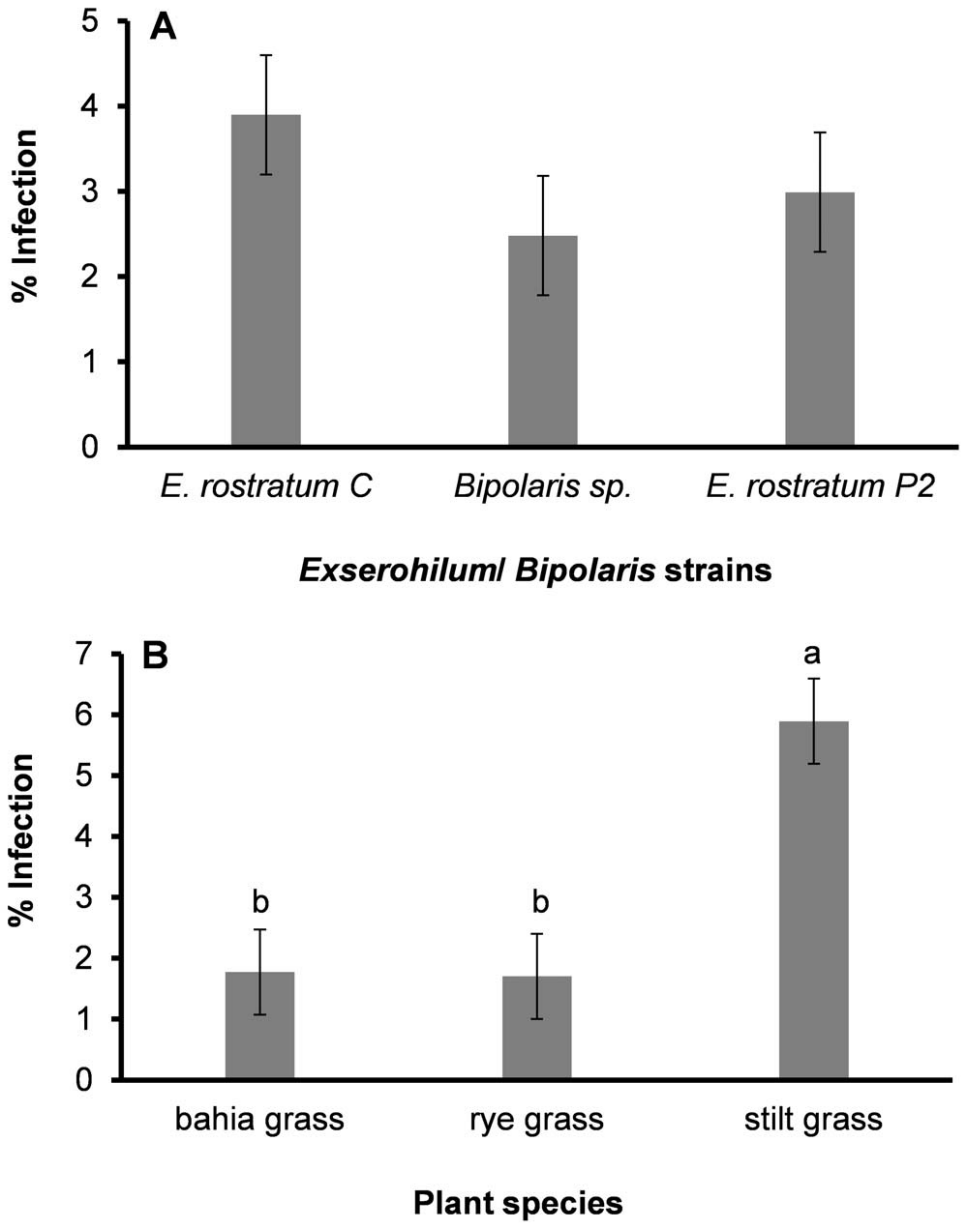
Effect of *Exserohilum* and *Bipolaris* isolates (A) and plant species (B) on % infection at six days after inoculation. Data are the means ± standard error of the second trial of the first experiment of the study, four replications (three pots per replication per treatment). There were no significant differences between the isolates of *Exserohilum* or *Bipolaris* at *P*<0.05. Plant species differed significantly in disease intensity (%) in reaction to isolates of *Exserohilum* or *Bipolaris*. Different letters above the bar indicate significant differences among plant species (*P* = 0.0003, Tukey test).

The number of pots with Exserohilum leaf spots out of the total number of pots with plants inoculated with *Exserohilum* and *Bipolaris* strains was greater in the second trial than in the first (Table 2). None of the plant species were resistant to any of the *Exserohilum* or *Bipolaris* isolates. Chi Square analysis of the number of pots with leaf spots out of the total number of pots with plants inoculated with *Exserohilum* or *Bipolaris* isolates indicated that stilt grass was the most and bahia grass the least susceptible to all isolates in both of the trials. Rye grass was as susceptible as stilt grass in the second trial, and as susceptible as bahia grass in the first trial. There were no differences in disease incidence between the fungal isolates in the first trial, but in the second trial the disease incidence by *E. rostratum* isolates was higher than by *Bipolaris* sp. In the second trial, percentages infection were not significantly different (F value  = 1.06, *P* = 0.36) among the *Exserohilum* and *Bipolaris* strains but differed significantly (F value  = 11.9, *P* = 0.0003) among plant species (Fig. 4). There was no significant interaction between isolates and plant species (F value  = 1.4, P = 0.2579). The mean percent infection was higher on stilt grass (% infection  = 5.9%) than on bahia grass and rye grass (% infection  = 1.8 and 1.7%, respectively).

**Table 2:** Number of pots with Exserohilum leaf spots out of the total number of pots with plants inoculated with strains of *Exserohilum* or *Bipolaris* at six days after inoculation.

Plant type	C3 plant	C3 plant	C4 plants	C4 plants	C4 plants	C4 plants	+/total strains	+/total strains	+/total strains
Grass species	Rye grass	Rye grass	Bahia grass	Bahia grass	Stilt grass	Stilt grass	All grasses	All grasses	All grasses
*Exserohilum* strains	Trial1	Trial2	Trial1	Trial2	Trial1	Trial2	Trial1	Trial2	Both trials
*E. rostratum* P2	2/10	11/12	2/10	10/12	6/6^3^	12/12	10/26	33/36 x^6^	43/62
*E. rostratum* C	3/10	12/12	4/9	9/12	6/6	12/12	13/25	33/36 x	46/61
*Bipolaris* sp.	1/10	12/12	3/10	5/12	6/6	12/12	10/26	29/36 y	39/62
Total	6/30 b^4^	35/36 A^5^	9/29 b	24/36 B	18/18 a	36/36 A	33/77	95/108	128/185

1 First trial had five replications (two pots per replication per treatment).

2 Second trial had four replications (three pots per replication per treatment).

3 Seeds of stilt grass had low germination; therefore, total number of plants are less for stilt grass than rye grass and bahia grass.

4 Different lower case letters indicate significant differences between plant species in the first trial (Chi^2^ tests  = 32.1, *P*<0.001).

5 Different upper case letters indicate significant differences between plant species in the second trial (Chi^2^ tests  = 23.3, *P*<0.001).

6 Different lower case letters indicate significant differences between pathogen strains in the second trial (Chi^2^ tests  = 84, *P*<0.001). No significant differences between the isolates were observed in the first trial.

**Figure 5:**
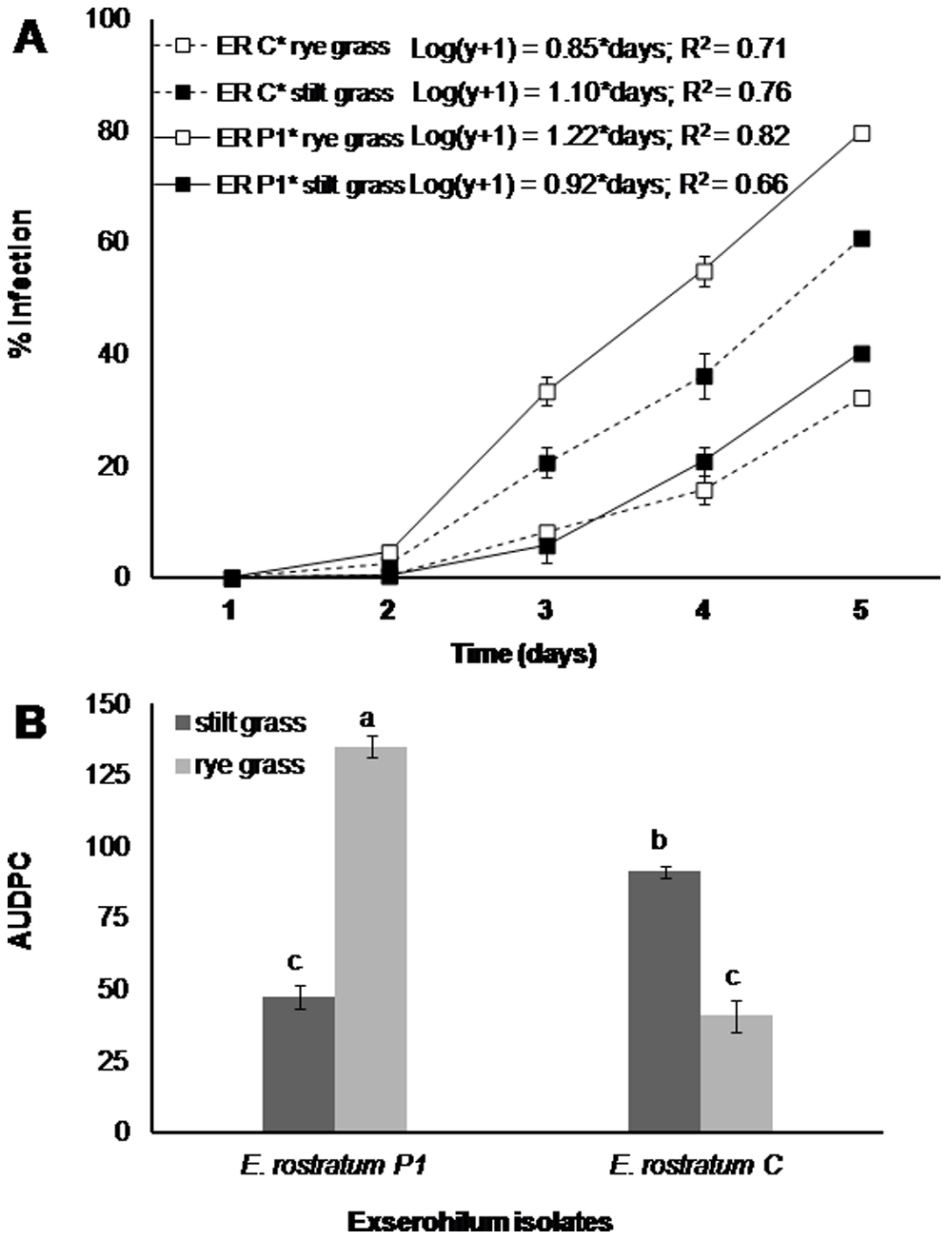
Reaction of stilt grass and rye grass to a clinical (ER C) and plant (ER P1) isolate of *Exserohilum rostratum* expressed as % infection over time (A) and area under disease progress curve (B) in the second experiment. Prior to regression analysis, diseased area data were log-transformed, but nontransformed means are presented in the figure for uniformity of presentations. Data are the means ± SE of two repetitions of the study, each with four replications (four per rep per treatment, five plants per pot). Significant differences are marked with different letters above bars (*P*≤0.05, Tukey-test).

In the second experiment, stilt grass and rye grass showed a differential response to the clinical and plant isolates of *E. rostratum*. Disease symptoms developed on both plant species two days after inoculation with both isolates of *E. rostratum* and increased over time. The increase of percent infection was exponential over time (Fig. 5a). The slope of the regression line of log-transformed data was significantly higher on rye grass inoculated with *E. rostratum* P1, intermediate on stilt grass inoculated with *E. rostratum* C, and least on stilt grass inoculated with *E. rostratum* P1 and rye grass inoculated with *E. rostratum* C (slope  = 1.22±0.015, 1.10±0.031, 0.76±0.031 and 0.71±0.071 log (%). day-1, respectively). Similar differences were obtained for AUDPC (area under disease progress curve) values. There was a highly significant interaction between plant species (stilt grass vs. rye grass) and isolate (*E*. *rostratum* C vs. P1) (P = 0.0001, F value  = 343.7). The mean AUDPCs in decreasing order were 135.1±4.1%. d on rye grass inoculated with *E. rostratum* P1, 91.2±1.9%. d on stilt grass inoculated with *E. rostratum* C, 47.4±4.1%. d on stilt grass inoculated with *E. rostratum* P1 and 40.7±5.7%. d on rye grass inoculated with *E. rostratum* C (Fig. 5b).

*Exserohilum* and *Bipolaris* strains were re-isolated by plating spores from infected tissues on PDA plates. After incubation for ten days, the re-isolated strains of *Exserohilum* and *Bipolaris* had the same colonies, as well as shape and size of conidia as observed for the initial isolates (Fig. 1).

## Discussion

This study confirmed that a clinical isolate from a deceased patient in Florida was *E. rostratum* based on colony and spore morphology as well as ITS and 28S DNA sequences, and was indistinguishable from a plant pathogenic isolate of *E. rostratum* that originated from tiger grass. The taxonomy and systematics of *Exserohilum* spp. are complex and many nomenclatural changes and refinements have been made [32], [40]. Many species of the genera *Bipolaris*, *Curvularia*, *Dissitimurus*, *Drechslera*, *Exserohilum* and *Helminthosporium* have been considered as synonyms [41], as they share most morphological characteristics [40]. However, there are many species of these genera that have not been sequenced and thus have not been assigned to any of the newly described genera [32]. Future studies using a polyphasic approach will be needed to refine the taxonomic position of the plant and clinical isolates of *Exserohilum* spp.

Besides the confirmation that a clinical and two plant pathogenic isolates of *E. rostratum* were morphologically and phylogenetically indistinguishable, the clinical isolate was shown to be at least as pathogenic to three grass species as the plant pathogenic isolates of *E. rostratum* and *Bipolaris sp*. *Exserohilum* species are common molds found in soil and on plants, especially grasses, and they thrive in humid climates. Some species can cause severe plant disease epidemics [31], [42]. *Exserohilum rostratum* has been recorded as a minor pathogen of humans, mainly in immunocompromised patients [29], but proved to be deadly when injected in the epidural space around the spinal column as a contaminant in a corticosteroid fluid [24]. The similarity in ribosomal DNA between the clinical and plant associated isolates of *E. rostratum* and the ability of the clinical isolate to infect plants suggest that the clinical *E. rostratum* isolate may have originated from plants. However, it remains unknown how this pathogen entered the compounding pharmacy in Massachusetts.

Two of the plant species that are susceptible to the clinical *E. rostratum* isolate, annual rye grass and Japanese stilt grass, are common in Massachusetts. *Exserohilum* spores are produced in moist environments on the surface of diseased leaves and they may become airborne [42]. *Exserohilum* spores from annual rye grass or Japanese stilt grass in the area around the compounding pharmacy might have entered this company via open windows or open air ducts and contaminated vials of the steroid solution. *Exserohilum* spores can also be carried over longer distances during wind storms [42]. Thus, *Exserohilum* spores could have entered the compounding pharmacy also as windborne spores from a long distance. Besides *E. rostratum*, several other environmental fungi (*Aspergillus* spp., *Cladosporium* spp., *Penicillium* spp.) were found in unopened vials of the steroid solutions [25]. Members of these genera can cause disease in plants or plant products [10], and produce large spore masses that are airborne [16]. Very few bacterial species were found in the vials, all closely related to *Bacillus* [25], indicating that the contamination was probably not due to dirty hands or water, but likely originated from the air.

Our research was carried out with a clinical isolate of *E. rostratum* that caused meningitis after injection with a contaminated steroid solution in the spinal cord of a patient who suffered from back pains. Because of the unusual route of infection, this case cannot be considered as a cross-kingdom adaptation of a pathogen. To be considered as such, a pathogen should be able to be transmitted among the new hosts or at least from the old to the new host via natural transmission routes. Other *E. rostratum* strains that caused various diseases via more natural transmission have been isolated from animals and humans [26], [27], [28], [29], but have not been tested for their ability to infect plants. However, these strains are likely also primarily plant-associated, suggesting that *E. rostratum* may have jumped from plants to the mammalian hosts. Additional clinical *E. rostratum* isolates would need to be tested for pathogenicity on plants to support this suggestion.

Several other plant pathogens that cause opportunistic human infections are transmitted to humans either by direct contact with the diseased plants, via other organisms like ants and flies, or via the air, water or food in nosocomial environments [26], [27], [28], [29]. For example, *Stenotrophomonas maltophilia* can be used as a biological control agent of *Ralstonia solanacearum*, the bacterial causal agent of potato brown rot [43], but is also associated with cystic fibrosis in humans [44]. Conversely, many human pathogens can be found in the rhizosphere of plants [12]. Species like *Salmonella enterica* and *Serratia marcescens* have clinical relevance and are able to enter plants via natural openings and wounds and multiply in the apoplast [45], [46], so that plants constitute a natural part of the microbial cycle [1], [2], [11]. Under conditions with reduced microbial competition these human pathogens can even induce symptoms on plants [47]. The ability of these cross-kingdom pathogens to maintain their population levels in a variety of niches increases the probability of emerging and re-emerging infectious diseases.

Cross-kingdom pathogens present opportunities for deciphering the mechanisms of host adaptation with respect to survival, attachment and infection in the plant rhizosphere and endosphere as well as the human or animal skin and intestinal tract [12]. Detailed information is already known about the infection processes of pathogenic bacteria in plants and animals, in particular with respect to the genetic determinants, effector secretion systems and/or toxin production [44], [47]. Much less information is available about infection mechanisms of fungi in animals compared to plants and about resistance factors in plants and animals [48]. The disease strategies used by cross-kingdom pathogens to infect unrelated hosts are interesting since animal and plant hosts have distinct physical barriers and defense responses [49].

Considering the fact that pathogens will continue to find ways to exploit novel host resources, future research activities should have a holistic approach to solving disease problems arising at the interface between plants, animals, humans and the ecosystems where they interact. A holistic approach to emerging infections and cross-kingdom host jumps should link microbiology, veterinary medicine, human medicine, ecology, public health, plant health, ecosystem health and epidemiology. The connections between animal health, human health and the environment have been embraced by the ‘One Health’ movement [50], [51] and a coordinated response to disease outbreaks promises to improve our capacity to deal more effectively with new emerging infectious diseases [52]. However, the ‘One Health’ concept does not thus far include plant health. Nevertheless, there is increasing evidence that health characteristics of different living components of ecosystems, including those dominated by humans, are connected and can be measured as the resilience of those components [53], [54], [55]. Evidence suggests that health of an ecosystem and its components is reduced at decreasing microbial diversity throughout the system [45], [54], [56]. The linkage between ecosystem health, soil health, plant health, animal health and human health likely comes about through the transfer of microbial communities through successive habitats and niches, as selected microbial communities are transferred together with the food that is consumed in complex food webs [2], [11], [54]. Diverse microbial communities are associated with induced systemic resistance in plants as well as enhanced immune systems in animals and humans [45], [50], [51]. We suggest that living components of ecosystems become more vulnerable to infection in stressed environments with reduced microbial diversities [57], [58]. This could have important implications for emerging infections by cross-kingdom pathogens with plants, animals and humans as potential hosts.

The findings from this study illustrate the complexity of the relationships between plant and human pathogens, and emphasize the need for surveillance of potential host-jumping events as an integrated part of human health care. The risk of host-jumping events is increased when natural host barriers are circumvented, for example by injections of contaminated corticosteroid solutions. The benefit of temporary pain relief may not outweigh the risk of unexpected infections [59] as exemplified by the outbreak of fungal meningitis in 2012. However, sanitary clinical environments alone cannot prevent the rise in cross-host infections. The maintenance of diverse and healthy ecosystems, including human dominated ecosystems, is key to stemming the emergence of pathogen adaptation to new hosts.

## Supporting Information

File S1 Supplementary Tables. **Table S1.** Raw data used for Table 2 in the main manuscript. The columns represent Experiment, Trial within experiment, Block within trial, pseudoreplicate within block, pathogen isolate, plant species, presence/absence of disease symptoms. ERP2  =  *Exserohilum rostratum* P2 (plant), ERC  =  *Exserohilum rostratum* C (clinical), Bipolaris  =  unidentified species of *Bipolaris*. **Table S2.** Raw data on spore measurements for *Exserohilum rostratum* C, *E. rostratum* P1 and P2 and *Bipolaris* sp. Columns represent spore number, pathogen isolate, condium length, conidium width; spore number, pathogen isolate, condium length, conidium width; spore number, pathogen isolate, condium length, conidium width; spore number, pathogen isolate, condium length, conidium width, for *Exserohilum rostratum* C, *E. rostratum* P1, *E. rostratum* P2, and *Bipolaris* sp., respectively. **Table S3.** Raw data used for Figure 4. The columns represent Experiment, Trial within experiment, Block within trial, pathogen isolate, plant species, and percent severity per plant. ERC  =  *Exserohilum rostratum* C (clinical), Bipolaris  =  unidentified species of *Bipolaris*, ERP2  =  *Exserohilum rostratum* P2 (plant). **Table S4.** Raw data used for Figure 5. The columns represent Experiment, Trial within experiment, Block within trial, pathogen isolate, plant species, pseudoreplication, average severity (%) per plant on day 2, area under the disease progress curve day 0–2, average severity (%) per plant on day 3, area under the disease progress curve day 2–3, average severity (%) per plant on day 4, area under the disease progress curve day 3–4, average severity (%) per plant on day 5, area under the disease progress curve day 4–5, and area under the disease progress curve day 0–5.
(DOCX)
