# Supplementary material for: Exserohilum rostratum: Characterization of a Cross-Kingdom Pathogen of Plants and Humans
Source: PLoS One. 2014 Oct 6;9(10):e108691. doi: 10.1371/journal.pone.0108691 (PMC4186819; doi:10.1371/journal.pone.0108691)
Supplement: File S1 — Supplementary Tables. Table S1. Raw data used for Table 2 in the main manuscript. The columns represent Experiment, Trial within experiment, Block within trial, pseudoreplicate within block, pathogen isolate, plant species, presence/absence of disease symptoms. ERP2 = Exserohilum rostratum P2 (plant), ERC = Exserohilum rostratum C (clinical), Bipolaris = unidentified species of Bipolaris. Table S2. Raw data on spore measurements for Exserohilum rostratum C, E. rostratum P1 and P2 and Bipolaris sp. Columns represent spore number, pathogen isolate, condium length, conidium width; spore number, pathogen isolate, condium length, conidium width; spore number, pathogen isolate, condium length, conidium width; spore number, pathogen isolate, condium length, conidium width, for Exserohilum rostratum C, E. rostratum P1, E. rostratum P2, and Bipolaris sp., respectively. Table S3. Raw data used for Figure 4. The columns represent Experiment, Trial within experiment, Block within trial, pathogen isolate, plant species, and percent severity per plant. ERC = Exserohilum rostratum C (clinical), Bipolaris = unidentified species of Bipolaris, ERP2 = Exserohilum rostratum P2 (plant). Table S4. Raw data used for Figure 5. The columns represent Experiment, Trial within experiment, Block within trial, pathogen isolate, plant species, pseudoreplication, average severity (%) per plant on day 2, area under the disease progress curve day 0–2, average severity (%) per plant on day 3, area under the disease progress curve day 2–3, average severity (%) per plant on day 4, area under the disease progress curve day 3–4, average severity (%) per plant on day 5, area under the disease progress curve day 4–5, and area under the disease progress curve day 0–5. (DOCX) [file pone.0108691.s001.docx]

Supplement for PONE-D-14-22677

***Exserohilum rostratum*: characterization of a cross-kingdom pathogen of plants and humans**

Kalpana Sharma^1^, Erica M. Goss^1,2^, Ellen R. Dickstein^1^, Matthew E. Smith^1^, Judith A. Johnson^2^, Frederick S. Southwick^3^, Ariena H.C. van Bruggen^1,2*^

^1^Department of Plant Pathology, IFAS, University of Florida, Gainesville, FL 32611, United States of America

^2^Emerging Pathogen Institute, University of Florida, Gainesville, FL 32610-0009, United States of America

^3^Department of Pathology, Immunology, and Laboratory Medicine, College of Medicine, Division of Infectious Diseases and Global Medicine, University of Florida, Gainesville, FL 32610-0277, United States of America

*Corresponding author: [ahcvanbruggen@ufl.edu](mailto:ahcvanbruggen@ufl.edu) (Ariena H.C. van Bruggen)

Table S1. Raw data used for Table 2 in the main manuscript. The columns represent Experiment, Trial within experiment, Block within trial, pseudoreplicate within block, pathogen isolate, plant species, presence / absence of disease symptoms. ERP2= *Exserohilum rostratum* P2 (plant), ERC=*Exserohilum rostratum* C (clinical), Bipolaris=unidentified species of *Bipolaris.*

| Exp | Trial | Block | pseudo | isolate | plant | yes/no |
| --- | --- | --- | --- | --- | --- | --- |
| 1 | 1 | 1 | A | ERP2 | bahia |  |
| 1 | 1 | 1 | A | ERP2 | rye |  |
| 1 | 1 | 1 | A | ERC | bahia | x |
| 1 | 1 | 1 | A | ERC | rye | x |
| 1 | 1 | 1 | A | Bipolaris | bahia |  |
| 1 | 1 | 1 | A | Bipolaris | rye |  |
| 1 | 1 | 1 | B | ERP2 | bahia |  |
| 1 | 1 | 1 | B | ERP2 | rye |  |
| 1 | 1 | 1 | B | ERC | bahia | x |
| 1 | 1 | 1 | B | ERC | rye | x |
| 1 | 1 | 1 | B | Bipolaris | bahia |  |
| 1 | 1 | 1 | B | Bipolaris | rye |  |
| 1 | 1 | 2 | A | ERP2 | bahia |  |
| 1 | 1 | 2 | A | ERP2 | rye |  |
| 1 | 1 | 2 | A | ERP2 | stilt grass | x |
| 1 | 1 | 2 | A | ERC | bahia |  |
| 1 | 1 | 2 | A | ERC | rye | x |
| 1 | 1 | 2 | A | ERC | stilt grass | x |
| 1 | 1 | 2 | A | Bipolaris | bahia |  |
| 1 | 1 | 2 | A | Bipolaris | rye |  |
| 1 | 1 | 2 | A | Bipolaris | stilt grass | x |
| 1 | 1 | 2 | B | ERP2 | bahia |  |
| 1 | 1 | 2 | B | ERP2 | rye |  |
| 1 | 1 | 2 | B | ERP2 | stilt grass | x |
| 1 | 1 | 2 | B | ERC | rye |  |
| 1 | 1 | 2 | B | ERC | stilt grass | x |
| 1 | 1 | 2 | B | ERC | bahia | x |
| 1 | 1 | 2 | B | Bipolaris | rye |  |
| 1 | 1 | 2 | B | Bipolaris | stilt grass | x |
| 1 | 1 | 3 | A | Bipolaris | bahia | x |
| 1 | 1 | 3 | A | ERP2 | rye | x |
| 1 | 1 | 3 | A | ERP2 | stilt grass | x |
| 1 | 1 | 3 | A | ERC | bahia |  |
| 1 | 1 | 3 | A | ERC | rye |  |
| 1 | 1 | 3 | A | ERC | stilt grass | x |
| 1 | 1 | 3 | A | Bipolaris | bahia | x |
| 1 | 1 | 3 | A | Bipolaris | rye |  |
| 1 | 1 | 3 | A | Bipolaris | stilt grass | x |
| 1 | 1 | 3 | B | ERP2 | bahia |  |
| 1 | 1 | 3 | B | ERP2 | rye |  |
| 1 | 1 | 3 | B | ERP2 | stilt grass | x |
| 1 | 1 | 3 | B | ERC | bahia | x |
| 1 | 1 | 3 | B | ERC | rye |  |
| 1 | 1 | 3 | B | ERC | stilt grass | x |
| 1 | 1 | 3 | B | Bipolaris | bahia |  |
| 1 | 1 | 3 | B | Bipolaris | rye |  |
| 1 | 1 | 3 | B | Bipolaris | Stilt grass | x |
| 1 | 1 | 4 | A | ERP2 | bahia | x |
| 1 | 1 | 4 | A | ERP2 | rye |  |
| 1 | 1 | 4 | A | ERC | bahia |  |
| 1 | 1 | 4 | A | ERC | rye |  |
| 1 | 1 | 4 | A | Bipolaris | bahia |  |
| 1 | 1 | 4 | A | Bipolaris | rye |  |
| 1 | 1 | 4 | B | ERP2 | bahia |  |
| 1 | 1 | 4 | B | ERP2 | rye |  |
| 1 | 1 | 4 | B | ERC | bahia |  |
| 1 | 1 | 4 | B | ERC | rye |  |
| 1 | 1 | 4 | B | Bipolaris | bahia |  |
| 1 | 1 | 4 | B | Bipolaris | rye |  |
| 1 | 1 | 5 | A | ERP2 | bahia |  |
| 1 | 1 | 5 | A | ERP2 | rye | x |
| 1 | 1 | 5 | A | ERP2 | stilt grass | x |
| 1 | 1 | 5 | A | ERC | bahia | x |
| 1 | 1 | 5 | A | ERC | rye |  |
| 1 | 1 | 5 | A | ERC | stilt grass | x |
| 1 | 1 | 5 | A | Bipolaris | bahia |  |
| 1 | 1 | 5 | A | Bipolaris | rye | x |
| 1 | 1 | 5 | A | Bipolaris | stilt grass | x |
| 1 | 1 | 5 | B | ERP2 | bahia |  |
| 1 | 1 | 5 | B | ERP2 | rye |  |
| 1 | 1 | 5 | B | ERP2 | stilt grass | x |
| 1 | 1 | 5 | B | ERC | bahia |  |
| 1 | 1 | 5 | B | ERC | rye |  |
| 1 | 1 | 5 | B | ERC | stilt grass | x |
| 1 | 1 | 5 | B | Bipolaris | bahia | x |
| 1 | 1 | 5 | B | Bipolaris | rye |  |
| 1 | 1 | 5 | B | Bipolaris | stilt grass | x |
| 1 | 2 | 1 | A | ERC | bahia | x |
| 1 | 2 | 1 | A | ERC | rye | x |
| 1 | 2 | 1 | A | ERC | stilt | x |
| 1 | 2 | 1 | A | Bipolaris | bahia |  |
| 1 | 2 | 1 | A | Bipolaris | rye | x |
| 1 | 2 | 1 | A | Bipolaris | stilt | x |
| 1 | 2 | 1 | A | ERP2 | bahia | x |
| 1 | 2 | 1 | A | ERP2 | rye | x |
| 1 | 2 | 1 | A | ERP2 | stilt | x |
| 1 | 2 | 1 | B | ERC | bahia | x |
| 1 | 2 | 1 | B | ERC | rye | x |
| 1 | 2 | 1 | B | ERC | stilt | x |
| 1 | 2 | 1 | B | Bipolaris | bahia | x |
| 1 | 2 | 1 | B | Bipolaris | rye | x |
| 1 | 2 | 1 | B | Bipolaris | stilt | x |
| 1 | 2 | 1 | B | ERP2 | bahia | x |
| 1 | 2 | 1 | B | ERP2 | rye | x |
| 1 | 2 | 1 | B | ERP2 | stilt | x |
| 1 | 2 | 1 | C | ERC | bahia |  |
| 1 | 2 | 1 | C | ERC | rye | x |
| 1 | 2 | 1 | C | ERC | stilt | x |
| 1 | 2 | 1 | C | Bipolaris | bahia | x |
| 1 | 2 | 1 | C | Bipolaris | rye | x |
| 1 | 2 | 1 | C | Bipolaris | stilt | x |
| 1 | 2 | 1 | C | ERP2 | bahia | x |
| 1 | 2 | 1 | C | ERP2 | rye | x |
| 1 | 2 | 1 | C | ERP2 | stilt | x |
| 1 | 2 | 2 | A | ERC | bahia | x |
| 1 | 2 | 2 | A | ERC | rye | x |
| 1 | 2 | 2 | A | ERC | stilt | x |
| 1 | 2 | 2 | A | Bipolaris | bahia |  |
| 1 | 2 | 2 | A | Bipolaris | rye | x |
| 1 | 2 | 2 | A | Bipolaris | stilt | x |
| 1 | 2 | 2 | A | ERP2 | bahia | x |
| 1 | 2 | 2 | A | ERP2 | rye | x |
| 1 | 2 | 2 | A | ERP2 | stilt | x |
| 1 | 2 | 2 | B | ERC | bahia | x |
| 1 | 2 | 2 | B | ERC | rye | x |
| 1 | 2 | 2 | B | ERC | stilt | x |
| 1 | 2 | 2 | B | Bipolaris | bahia |  |
| 1 | 2 | 2 | B | Bipolaris | rye | x |
| 1 | 2 | 2 | B | Bipolaris | stilt | x |
| 1 | 2 | 2 | B | ERP2 | bahia |  |
| 1 | 2 | 2 | B | ERP2 | rye | x |
| 1 | 2 | 2 | B | ERP2 | stilt | x |
| 1 | 2 | 2 | C | ERC | bahia | x |
| 1 | 2 | 2 | C | ERC | rye | x |
| 1 | 2 | 2 | C | ERC | stilt | x |
| 1 | 2 | 2 | C | Bipolaris | bahia | x |
| 1 | 2 | 2 | C | Bipolaris | rye | x |
| 1 | 2 | 2 | C | Bipolaris | stilt | x |
| 1 | 2 | 2 | C | ERP2 | bahia | x |
| 1 | 2 | 2 | C | ERP2 | rye |  |
| 1 | 2 | 2 | C | ERP2 | stilt | x |
| 1 | 2 | 3 | A | ERC | bahia | x |
| 1 | 2 | 3 | A | ERC | rye | x |
| 1 | 2 | 3 | A | ERC | stilt | x |
| 1 | 2 | 3 | A | Bipolaris | bahia | x |
| 1 | 2 | 3 | A | Bipolaris | rye | x |
| 1 | 2 | 3 | A | Bipolaris | stilt | x |
| 1 | 2 | 3 | A | ERP2 | bahia | x |
| 1 | 2 | 3 | A | ERP2 | rye | x |
| 1 | 2 | 3 | A | ERP2 | stilt | x |
| 1 | 2 | 3 | B | ERC | bahia | x |
| 1 | 2 | 3 | B | ERC | rye | x |
| 1 | 2 | 3 | B | ERC | stilt | x |
| 1 | 2 | 3 | B | Bipolaris | bahia |  |
| 1 | 2 | 3 | B | Bipolaris | rye | x |
| 1 | 2 | 3 | B | Bipolaris | stilt | x |
| 1 | 2 | 3 | B | ERP2 | bahia | x |
| 1 | 2 | 3 | B | ERP2 | rye | x |
| 1 | 2 | 3 | B | ERP2 | stilt | x |
| 1 | 2 | 3 | C | ERC | bahia | x |
| 1 | 2 | 3 | C | ERC | rye | x |
| 1 | 2 | 3 | C | ERC | stilt | x |
| 1 | 2 | 3 | C | Bipolaris | bahia |  |
| 1 | 2 | 3 | C | Bipolaris | rye | x |
| 1 | 2 | 3 | C | Bipolaris | stilt | x |
| 1 | 2 | 3 | C | ERP2 | bahia | x |
| 1 | 2 | 3 | C | ERP2 | rye | x |
| 1 | 2 | 3 | C | ERP2 | stilt | x |
| 1 | 2 | 4 | A | ERC | bahia |  |
| 1 | 2 | 4 | A | ERC | rye | x |
| 1 | 2 | 4 | A | ERC | stilt | x |
| 1 | 2 | 4 | A | Bipolaris | bahia |  |
| 1 | 2 | 4 | A | Bipolaris | rye | x |
| 1 | 2 | 4 | A | Bipolaris | stilt | x |
| 1 | 2 | 4 | A | ERP2 | bahia |  |
| 1 | 2 | 4 | A | ERP2 | rye | x |
| 1 | 2 | 4 | A | ERP2 | stilt | x |
| 1 | 2 | 4 | B | ERC | bahia |  |
| 1 | 2 | 4 | B | ERC | rye | x |
| 1 | 2 | 4 | B | ERC | stilt | x |
| 1 | 2 | 4 | B | Bipolaris | bahia | x |
| 1 | 2 | 4 | B | Bipolaris | rye | x |
| 1 | 2 | 4 | B | Bipolaris | stilt | x |
| 1 | 2 | 4 | B | ERP2 | bahia | x |
| 1 | 2 | 4 | B | ERP2 | rye | x |
| 1 | 2 | 4 | B | ERP2 | stilt | x |
| 1 | 2 | 4 | C | ERC | bahia | x |
| 1 | 2 | 4 | C | ERC | rye | x |
| 1 | 2 | 4 | C | ERC | stilt | x |
| 1 | 2 | 4 | C | Bipolaris | bahia |  |
| 1 | 2 | 4 | C | Bipolaris | rye | x |
| 1 | 2 | 4 | C | Bipolaris | stilt | x |
| 1 | 2 | 4 | C | ERP2 | bahia | x |
| 1 | 2 | 4 | C | ERP2 | rye | x |
| 1 | 2 | 4 | C | ERP2 | stilt | x |

Table S2. Raw data on spore measurements for *Exserohilum rostratum* C, *E. rostratum* P1 and P2 and *Bipolaris* sp. Columns represent spore number, pathogen isolate, conidium length, conidium width; spore number, pathogen isolate, conidium length, conidium width;

spore number, pathogen isolate, conidium length, conidium width; spore number, pathogen isolate, conidium length, conidium width, for *Exserohilum rostratum* C, *E. rostratum* P1, *E. rostratum* P2, and *Bipolaris* sp., respectively.

| SN | Isol. | Length | Width | SN | Isol. | Length | Width | SN | Isol. | Length | Width | SN | Isol. | Length | Width |
| --- | --- | --- | --- | --- | --- | --- | --- | --- | --- | --- | --- | --- | --- | --- | --- |
| 1 | ER C | 20.0 | 11.0 | 1 | ER P1 | 44.1 | 17.5 | 1 | ER P2 | 15.3 | 13.3 | 1 | Bip. | 18.3 | 9.8 |
| 2 | ER C | 23.0 | 19.4 | 2 | ER P1 | 20.3 | 12.2 | 2 | ER P2 | 16.4 | 13.0 | 2 | Bip. | 20.4 | 9.3 |
| 3 | ER C | 34.3 | 16.2 | 3 | ER P1 | 21.5 | 18.6 | 3 | ER P2 | 15.9 | 13.2 | 3 | Bip. | 20.5 | 8.3 |
| 4 | ER C | 33.2 | 17.5 | 4 | ER P1 | 65.5 | 11.5 | 4 | ER P2 | 20.0 | 18.0 | 4 | Bip. | 20.0 | 9.4 |
| 5 | ER C | 25.7 | 18.9 | 5 | ER P1 | 25.0 | 15.3 | 5 | ER P2 | 15.0 | 13.4 | 5 | Bip. | 8.0 | 9.3 |
| 6 | ER C | 27.0 | 13.5 | 6 | ER P1 | 27.6 | 16.6 | 6 | ER P2 | 16.0 | 13.0 | 6 | Bip. | 9.8 | 8.9 |
| 7 | ER C | 29.8 | 15.1 | 7 | ER P1 | 30.5 | 11.0 | 7 | ER P2 | 18.0 | 13.4 | 7 | Bip. | 33.0 | 9.3 |
| 8 | ER C | 30.3 | 16.4 | 8 | ER P1 | 37.7 | 20.0 | 8 | ER P2 | 22.8 | 13.3 | 8 | Bip. | 22.8 | 9.2 |
| 9 | ER C | 33.0 | 19.5 | 9 | ER P1 | 33.9 | 20.0 | 9 | ER P2 | 16.0 | 13.9 | 9 | Bip. | 26.5 | 9.8 |
| 10 | ER C | 51.5 | 19.1 | 10 | ER P1 | 20.0 | 19.8 | 10 | ER P2 | 16.3 | 13.1 | 10 | Bip. | 24.3 | 9.0 |
| 11 | ER C | 45.0 | 12.0 | 11 | ER P1 | 37.0 | 15.7 | 11 | ER P2 | 18.0 | 13.3 | 11 | Bip. | 8.0 | 9.2 |
| 12 | ER C | 45.3 | 13.7 | 12 | ER P1 | 22.8 | 14.9 | 12 | ER P2 | 15.0 | 13.4 | 12 | Bip. | 26.0 | 9.3 |
| 13 | ER C | 23.0 | 15.1 | 13 | ER P1 | 33.5 | 14.3 | 13 | ER P2 | 17.8 | 13.3 | 13 | Bip. | 20.0 | 9.5 |
| 14 | ER C | 44.8 | 16.4 | 14 | ER P1 | 35.3 | 15.6 | 14 | ER P2 | 21.4 | 13.6 | 14 | Bip. | 22.3 | 6.4 |
| 15 | ER C | 23.6 | 17.6 | 15 | ER P1 | 28.1 | 18.5 | 15 | ER P2 | 17.0 | 13.2 | 15 | Bip. | 20.0 | 10.0 |
| 16 | ER C | 24.3 | 20.0 | 16 | ER P1 | 29.8 | 17.0 | 16 | ER P2 | 21.5 | 13.4 | 16 | Bip. | 30.1 | 9.3 |
| 17 | ER C | 34.0 | 18.5 | 17 | ER P1 | 22.6 | 20.0 | 17 | ER P2 | 17.0 | 13.2 | 17 | Bip. | 22.4 | 9.1 |
| 18 | ER C | 65.5 | 15.6 | 18 | ER P1 | 24.3 | 19.9 | 18 | ER P2 | 18.5 | 13.3 | 18 | Bip. | 23.7 | 9.5 |
| 19 | ER C | 25.7 | 19.3 | 19 | ER P1 | 23.5 | 17.3 | 19 | ER P2 | 15.9 | 13.2 | 19 | Bip. | 20.0 | 9.4 |
| 20 | ER C | 25.2 | 16.4 | 20 | ER P1 | 30.0 | 18.2 | 20 | ER P2 | 20.4 | 13.3 | 20 | Bip. | 25.5 | 9.2 |
| 21 | ER C | 26.1 | 14.8 | 21 | ER P1 | 33.0 | 15.8 | 21 | ER P2 | 18.0 | 13.5 | 21 | Bip. | 20.0 | 9.4 |
| 22 | ER C | 27.4 | 11.8 | 22 | ER P1 | 22.8 | 16.0 | 22 | ER P2 | 15.6 | 13.3 | 22 | Bip. | 23.8 | 9.2 |
| 23 | ER C | 27.3 | 14.6 | 23 | ER P1 | 38.0 | 15.0 | 23 | ER P2 | 15.4 | 13.3 | 23 | Bip. | 25.0 | 9.2 |
| 24 | ER C | 32.7 | 15.5 | 24 | ER P1 | 24.6 | 14.5 | 24 | ER P2 | 18.6 | 13.0 | 24 | Bip. | 24.7 | 8.9 |
| 25 | ER C | 30.0 | 14.3 | 25 | ER P1 | 26.4 | 15.3 | 25 | ER P2 | 16.7 | 13.1 | 25 | Bip. | 19.3 | 10.0 |
| 26 | ER C | 28.0 | 16.2 | 26 | ER P1 | 30.0 | 14.7 | 26 | ER P2 | 22.7 | 13.2 | 26 | Bip. | 20.6 | 9.1 |
| 27 | ER C | 29.0 | 15.2 | 27 | ER P1 | 24.0 | 11.8 | 27 | ER P2 | 18.1 | 13.0 | 27 | Bip. | 20.8 | 8.9 |
| 28 | ER C | 30.0 | 18.1 | 28 | ER P1 | 29.0 | 14.6 | 28 | ER P2 | 17.0 | 13.6 | 28 | Bip. | 20.6 | 9.5 |
| 29 | ER C | 22.8 | 20.0 | 29 | ER P1 | 23.1 | 19.8 | 29 | ER P2 | 18.0 | 13.3 | 29 | Bip. | 33.0 | 9.2 |
| 30 | ER C | 21.6 | 16.3 | 30 | ER P1 | 24.6 | 16.8 | 30 | ER P2 | 16.3 | 13.7 | 30 | Bip. | 21.2 | 9.6 |
| 31 | ER C | 23.3 | 15.5 | 31 | ER P1 | 25.8 | 16.0 | 31 | ER P2 | 16.5 | 13.0 | 31 | Bip. | 20.6 | 8.9 |
| 32 | ER C | 30.5 | 17.4 | 32 | ER P1 | 27.0 | 13.8 | 32 | ER P2 | 17.4 | 13.1 | 32 | Bip. | 30.2 | 9.0 |
| 33 | ER C | 23.0 | 18.0 | 33 | ER P1 | 25.6 | 20.0 | 33 | ER P2 | 18.0 | 13.0 | 33 | Bip. | 21.3 | 8.9 |
| 34 | ER C | 22.7 | 14.4 | 34 | ER P1 | 40.1 | 18.2 | 34 | ER P2 | 20.1 | 13.0 | 34 | Bip. | 20.1 | 9.8 |
| 35 | ER C | 24.0 | 14.2 | 35 | ER P1 | 25.0 | 17.7 | 35 | ER P2 | 16.2 | 13.4 | 35 | Bip. | 23.5 | 9.3 |
| 36 | ER C | 29.5 | 20.0 | 36 | ER P1 | 24.8 | 15.0 | 36 | ER P2 | 21.0 | 13.1 | 36 | Bip. | 21.0 | 9.0 |
| 37 | ER C | 30.0 | 18.2 | 37 | ER P1 | 32.7 | 15.5 | 37 | ER P2 | 16.7 | 13.0 | 37 | Bip. | 20.0 | 9.5 |
| 38 | ER C | 30.5 | 15.5 | 38 | ER P1 | 50.1 | 14.0 | 38 | ER P2 | 15.1 | 13.3 | 38 | Bip. | 19.4 | 9.2 |
| 39 | ER C | 31.7 | 16.7 | 39 | ER P1 | 28.0 | 14.1 | 39 | ER P2 | 19.3 | 13.1 | 39 | Bip. | 22.2 | 9.0 |
| 40 | ER C | 31.3 | 16.5 | 40 | ER P1 | 37.5 | 14.2 | 40 | ER P2 | 20.1 | 13.0 | 40 | Bip. | 23.5 | 9.9 |
| 41 | ER C | 34.5 | 15.2 | 41 | ER P1 | 24.9 | 15.6 | 41 | ER P2 | 16.0 | 13.2 | 41 | Bip. | 24.4 | 9.1 |
| 42 | ER C | 28.6 | 15.4 | 42 | ER P1 | 30.0 | 13.5 | 42 | ER P2 | 15.4 | 13.1 | 42 | Bip. | 23.7 | 9.0 |
| 43 | ER C | 30.0 | 16.6 | 43 | ER P1 | 35.5 | 14.4 | 43 | ER P2 | 15.0 | 13.0 | 43 | Bip. | 22.1 | 8.9 |
| 44 | ER C | 29.3 | 15.9 | 44 | ER P1 | 26.3 | 12.6 | 44 | ER P2 | 19.0 | 13.0 | 44 | Bip. | 20.0 | 9.0 |
| 45 | ER C | 27.3 | 14.5 | 45 | ER P1 | 25.7 | 19.8 | 45 | ER P2 | 44.0 | 13.0 | 45 | Bip. | 25.0 | 8.9 |
| 46 | ER C | 25.1 | 17.0 | 46 | ER P1 | 32.8 | 18.6 | 46 | ER P2 | 18.0 | 13.1 | 46 | Bip. | 24.6 | 9.7 |
| 47 | ER C | 26.4 | 12.7 | 47 | ER P1 | 38.5 | 20.0 | 47 | ER P2 | 15.0 | 13.0 | 47 | Bip. | 22.3 | 8.9 |
| 48 | ER C | 25.0 | 11.5 | 48 | ER P1 | 30.0 | 12.0 | 48 | ER P2 | 16.0 | 13.1 | 48 | Bip. | 21.5 | 10.0 |
| 49 | ER C | 28.6 | 14.5 | 49 | ER P1 | 27.3 | 13.0 | 49 | ER P2 | 16.4 | 13.0 | 49 | Bip. | 24.2 | 8.9 |
| 50 | ER C | 30.0 | 12.8 | 50 | ER P1 | 25.8 | 14.2 | 50 | ER P2 | 15.2 | 13.2 | 50 | Bip. | 22.5 | 9.1 |

Table S3. Raw data used for Figure 4. The columns represent Experiment, Trial within experiment, Block within trial, pathogen isolate, plant species, and percent severity per plant. ERC=*Exserohilum rostratum* C (clinical), Bipolaris=unidentified species of *Bipolaris,* ERP2= *Exserohilum rostratum* P2 (plant).

| EXP | Trial | Block | isolate | species | sev (%) |
| --- | --- | --- | --- | --- | --- |
| 1 | 2 | 1 | ERC | bahia | 5.70 |
| 1 | 2 | 1 | ERC | rye | 1.04 |
| 1 | 2 | 1 | ERC | stilt | 9.20 |
| 1 | 2 | 1 | Bipolaris | bahia | 1.11 |
| 1 | 2 | 1 | Bipolaris | rye | 3.33 |
| 1 | 2 | 1 | Bipolaris | stilt | 1.93 |
| 1 | 2 | 1 | ERP2 | bahia | 0.57 |
| 1 | 2 | 1 | ERP2 | rye | 0.56 |
| 1 | 2 | 1 | ERP2 | stilt | 11.53 |
| 1 | 2 | 2 | ERC | bahia | 1.78 |
| 1 | 2 | 2 | ERC | rye | 3.28 |
| 1 | 2 | 2 | ERC | stilt | 2.29 |
| 1 | 2 | 2 | Bipolaris | bahia | 1.83 |
| 1 | 2 | 2 | Bipolaris | rye | 2.18 |
| 1 | 2 | 2 | Bipolaris | stilt | 6.53 |
| 1 | 2 | 2 | ERP2 | bahia | 1.05 |
| 1 | 2 | 2 | ERP2 | rye | 0.97 |
| 1 | 2 | 2 | ERP2 | stilt | 4.46 |
| 1 | 2 | 3 | ERC | bahia | 0.84 |
| 1 | 2 | 3 | ERC | rye | 0.84 |
| 1 | 2 | 3 | ERC | stilt | 8.97 |
| 1 | 2 | 3 | Bipolaris | bahia | 0.17 |
| 1 | 2 | 3 | Bipolaris | rye | 3.25 |
| 1 | 2 | 3 | Bipolaris | stilt | 4.87 |
| 1 | 2 | 3 | ERP2 | bahia | 4.99 |
| 1 | 2 | 3 | ERP2 | rye | 0.76 |
| 1 | 2 | 3 | ERP2 | stilt | 2.09 |
| 1 | 2 | 4 | ERC | bahia | 0.11 |
| 1 | 2 | 4 | ERC | rye | 1.38 |
| 1 | 2 | 4 | ERC | stilt | 11.33 |
| 1 | 2 | 4 | Bipolaris | bahia | 0.67 |
| 1 | 2 | 4 | Bipolaris | rye | 1.96 |
| 1 | 2 | 4 | Bipolaris | stilt | 1.93 |
| 1 | 2 | 4 | ERP2 | bahia | 2.44 |
| 1 | 2 | 4 | ERP2 | rye | 0.88 |
| 1 | 2 | 4 | ERP2 | stilt | 5.58 |

Table S4. Raw data used for Figure 5. The columns represent Experiment, Trial within experiment, Block within trial, pathogen isolate, plant species, pseudoreplication, average severity (%) per plant on day 2, area under the disease progress curve day 0-2, average severity (%) per plant on day 3, area under the disease progress curve day 2-3, average severity (%) per plant on day 4, area under the disease progress curve day 3-4, average severity (%) per plant on day 5, area under the disease progress curve day 4-5, and area under the disease progress curve day 0-5.

| Exp. | Trial | Block | Isolate | species | pseudo | sev(%) 2 | AUD2 | sev(%) 3 | AUD3 | sev(%) 4 | AUD4 | sev(%) 5 | AUD5 | TAUD |
| --- | --- | --- | --- | --- | --- | --- | --- | --- | --- | --- | --- | --- | --- | --- |
| 2 | 1 | 1 | ERP1 | Rye | 1 | 5 | 5 | 30 | 17.5 | 49 | 39.5 | 70 | 59.5 | 121.5 |
| 2 | 1 | 1 | ERP1 | Rye | 2 | 3 | 3 | 33 | 18 | 52 | 42.5 | 75 | 63.5 | 127 |
| 2 | 1 | 1 | ERP1 | Rye | 3 | 3 | 3 | 28 | 15.5 | 52 | 40 | 72 | 62 | 120.5 |
| 2 | 1 | 1 | ERP1 | Rye | 4 | 4 | 4 | 31 | 17.5 | 54 | 42.5 | 74 | 64 | 128 |
| 2 | 1 | 1 | ERP1 | Stilt | 1 | 1 | 1 | 5 | 3 | 12 | 8.5 | 30 | 21 | 33.5 |
| 2 | 1 | 1 | ERP1 | Stilt | 2 | 0.5 | 0.5 | 6 | 3.25 | 13 | 9.5 | 32 | 22.5 | 35.75 |
| 2 | 1 | 1 | ERP1 | Stilt | 3 | 1 | 1 | 3 | 2 | 14 | 8.5 | 35 | 24.5 | 36 |
| 2 | 1 | 1 | ERP1 | Stilt | 4 | 0.7 | 0.7 | 5 | 2.85 | 11 | 8 | 31 | 21 | 32.55 |
| 2 | 1 | 1 | ERC | Rye | 1 | 0.1 | 0.1 | 7 | 3.55 | 20 | 13.5 | 30 | 25 | 42.15 |
| 2 | 1 | 1 | ERC | Rye | 2 | 0.2 | 0.2 | 6 | 3.1 | 18 | 12 | 28 | 23 | 38.3 |
| 2 | 1 | 1 | ERC | Rye | 3 | 0.1 | 0.1 | 4 | 2.05 | 19 | 11.5 | 29 | 24 | 37.65 |
| 2 | 1 | 1 | ERC | Rye | 4 | 0.2 | 0.2 | 8 | 4.1 | 21 | 14.5 | 35 | 28 | 46.8 |
| 2 | 1 | 1 | ERC | Stilt | 1 | 2 | 2 | 15 | 8.5 | 20 | 17.5 | 45 | 32.5 | 60.5 |
| 2 | 1 | 1 | ERC | Stilt | 2 | 1 | 1 | 12 | 6.5 | 18 | 15 | 40 | 29 | 51.5 |
| 2 | 1 | 1 | ERC | Stilt | 3 | 2 | 2 | 13 | 7.5 | 22 | 17.5 | 44 | 33 | 60 |
| 2 | 1 | 1 | ERC | Stilt | 4 | 2.5 | 2.5 | 16 | 9.25 | 20 | 18 | 42 | 31 | 60.75 |
| 2 | 1 | 2 | ERP1 | Rye | 1 | 4 | 4 | 26 | 15 | 50 | 38 | 70 | 60 | 117 |
| 2 | 1 | 2 | ERP1 | Rye | 2 | 6 | 6 | 28 | 17 | 58 | 43 | 80 | 69 | 135 |
| 2 | 1 | 2 | ERP1 | Rye | 3 | 8 | 8 | 33 | 20.5 | 60 | 46.5 | 85 | 72.5 | 147.5 |
| 2 | 1 | 2 | ERP1 | Rye | 4 | 5 | 5 | 30 | 17.5 | 55 | 42.5 | 75 | 65 | 130 |
| 2 | 1 | 2 | ERP1 | Stilt | 1 | 0.2 | 0.2 | 7 | 3.6 | 15 | 11 | 30 | 22.5 | 37.3 |
| 2 | 1 | 2 | ERP1 | Stilt | 2 | 0.3 | 0.3 | 8 | 4.15 | 13 | 10.5 | 40 | 26.5 | 41.45 |
| 2 | 1 | 2 | ERP1 | Stilt | 3 | 0.5 | 0.5 | 8 | 4.25 | 14 | 11 | 37 | 25.5 | 41.25 |
| 2 | 1 | 2 | ERP1 | Stilt | 4 | 0.2 | 0.2 | 7 | 3.6 | 18 | 12.5 | 35 | 26.5 | 42.8 |
| 2 | 1 | 2 | ERC | Rye | 1 | 0.3 | 0.3 | 6 | 3.15 | 15 | 10.5 | 30 | 22.5 | 36.45 |
| 2 | 1 | 2 | ERC | Rye | 2 | 0.2 | 0.2 | 8 | 4.1 | 13 | 10.5 | 24 | 18.5 | 33.3 |
| 2 | 1 | 2 | ERC | Rye | 3 | 0.1 | 0.1 | 7.5 | 3.8 | 12 | 9.75 | 20 | 16 | 29.65 |
| 2 | 1 | 2 | ERC | Rye | 4 | 0.2 | 0.2 | 7 | 3.6 | 12 | 9.5 | 22 | 17 | 30.3 |
| 2 | 1 | 2 | ERC | Stilt | 1 | 2 | 2 | 17 | 9.5 | 35 | 26 | 60 | 47.5 | 85 |
| 2 | 1 | 2 | ERC | Stilt | 2 | 4 | 4 | 20 | 12 | 40 | 30 | 65 | 52.5 | 98.5 |
| 2 | 1 | 2 | ERC | Stilt | 3 | 3 | 3 | 18 | 10.5 | 36 | 27 | 70 | 53 | 93.5 |
| 2 | 1 | 2 | ERC | Stilt | 4 | 2.7 | 2.7 | 19.5 | 11.1 | 39 | 29.25 | 64 | 51.5 | 94.55 |
| 2 | 1 | 3 | ERP1 | Rye | 1 | 4 | 4 | 37 | 20.5 | 48 | 42.5 | 70 | 59 | 126 |
| 2 | 1 | 3 | ERP1 | Rye | 2 | 5 | 5 | 33 | 19 | 45 | 39 | 72 | 58.5 | 121.5 |
| 2 | 1 | 3 | ERP1 | Rye | 3 | 4 | 4 | 35 | 19.5 | 47 | 41 | 68 | 57.5 | 122 |
| 2 | 1 | 3 | ERP1 | Rye | 4 | 5 | 5 | 45 | 25 | 53 | 49 | 73 | 63 | 142 |
| 2 | 1 | 3 | ERP1 | Stilt | 1 | 0.3 | 0.3 | 8 | 4.15 | 22 | 15 | 40 | 31 | 50.45 |
| 2 | 1 | 3 | ERP1 | Stilt | 2 | 0.4 | 0.4 | 9 | 4.7 | 25 | 17 | 38 | 31.5 | 53.6 |
| 2 | 1 | 3 | ERP1 | Stilt | 3 | 0.3 | 0.3 | 9.5 | 4.9 | 24 | 16.75 | 35 | 29.5 | 51.45 |
| 2 | 1 | 3 | ERP1 | Stilt | 4 | 1 | 1 | 12 | 6.5 | 26 | 19 | 37 | 31.5 | 58 |
| 2 | 1 | 3 | ERC | Rye | 1 | 0.5 | 0.5 | 8 | 4.25 | 12 | 10 | 25 | 18.5 | 33.25 |
| 2 | 1 | 3 | ERC | Rye | 2 | 0.8 | 0.8 | 9 | 4.9 | 14 | 11.5 | 24 | 19 | 36.2 |
| 2 | 1 | 3 | ERC | Rye | 3 | 0.3 | 0.3 | 10 | 5.15 | 15 | 12.5 | 22 | 18.5 | 36.45 |
| 2 | 1 | 3 | ERC | Rye | 4 | 0.2 | 0.2 | 9 | 4.6 | 13 | 11 | 24 | 18.5 | 34.3 |
| 2 | 1 | 3 | ERC | Stilt | 1 | 2 | 2 | 20 | 11 | 30 | 25 | 52 | 41 | 79 |
| 2 | 1 | 3 | ERC | Stilt | 2 | 2 | 2 | 21 | 11.5 | 35 | 28 | 58 | 46.5 | 88 |
| 2 | 1 | 3 | ERC | Stilt | 3 | 1.5 | 1.5 | 18 | 9.75 | 30 | 24 | 53 | 41.5 | 76.75 |
| 2 | 1 | 3 | ERC | Stilt | 4 | 2 | 2 | 19 | 10.5 | 38 | 28.5 | 55 | 46.5 | 87.5 |
| 2 | 1 | 4 | ERP1 | Rye | 1 | 5 | 5 | 32 | 18.5 | 75 | 53.5 | 98 | 86.5 | 163.5 |
| 2 | 1 | 4 | ERP1 | Rye | 2 | 6 | 6 | 38 | 22 | 65 | 51.5 | 95 | 80 | 159.5 |
| 2 | 1 | 4 | ERP1 | Rye | 3 | 5 | 5 | 40 | 22.5 | 73 | 56.5 | 85 | 79 | 163 |
| 2 | 1 | 4 | ERP1 | Rye | 4 | 4 | 4 | 37 | 20.5 | 75 | 56 | 90 | 82.5 | 163 |
| 2 | 1 | 4 | ERP1 | Stilt | 1 | 0.3 | 0.3 | 4 | 2.15 | 17 | 10.5 | 45 | 31 | 43.95 |
| 2 | 1 | 4 | ERP1 | Stilt | 2 | 1 | 1 | 3 | 2 | 28 | 15.5 | 48 | 38 | 56.5 |
| 2 | 1 | 4 | ERP1 | Stilt | 3 | 0.5 | 0.5 | 4 | 2.25 | 20 | 12 | 53 | 36.5 | 51.25 |
| 2 | 1 | 4 | ERP1 | Stilt | 4 | 0.8 | 0.8 | 4.5 | 2.65 | 20 | 12.25 | 55 | 37.5 | 53.2 |
| 2 | 1 | 4 | ERC | Rye | 1 | 0.3 | 0.3 | 8 | 4.15 | 15 | 11.5 | 45 | 30 | 45.95 |
| 2 | 1 | 4 | ERC | Rye | 2 | 0.2 | 0.2 | 9 | 4.6 | 18 | 13.5 | 50 | 34 | 52.3 |
| 2 | 1 | 4 | ERC | Rye | 3 | 0.4 | 0.4 | 8.5 | 4.45 | 16 | 12.25 | 48 | 32 | 49.1 |
| 2 | 1 | 4 | ERC | Rye | 4 | 0.3 | 0.3 | 7 | 3.65 | 15 | 11 | 44 | 29.5 | 44.45 |
| 2 | 1 | 4 | ERC | Stilt | 1 | 3 | 3 | 19 | 11 | 38 | 28.5 | 70 | 54 | 96.5 |
| 2 | 1 | 4 | ERC | Stilt | 2 | 4 | 4 | 21 | 12.5 | 32 | 26.5 | 65 | 48.5 | 91.5 |
| 2 | 1 | 4 | ERC | Stilt | 3 | 3.5 | 3.5 | 22 | 12.75 | 36 | 29 | 70 | 53 | 98.25 |
| 2 | 1 | 4 | ERC | Stilt | 4 | 4 | 4 | 20 | 12 | 35 | 27.5 | 68 | 51.5 | 95 |
| 2 | 2 | 1 | ERP1 | Rye | 1 | 3 | 3 | 35 | 19 | 50 | 42.5 | 88 | 69 | 133.5 |
| 2 | 2 | 1 | ERP1 | Rye | 2 | 6 | 6 | 37 | 21.5 | 48 | 42.5 | 75 | 61.5 | 131.5 |
| 2 | 2 | 1 | ERP1 | Rye | 3 | 5 | 5 | 40 | 22.5 | 50 | 45 | 82 | 66 | 138.5 |
| 2 | 2 | 1 | ERP1 | Rye | 4 | 4 | 4 | 36 | 20 | 55 | 45.5 | 83 | 69 | 138.5 |
| 2 | 2 | 1 | ERP1 | Stilt | 1 | 0 | 0 | 3 | 1.5 | 28 | 15.5 | 44 | 36 | 53 |
| 2 | 2 | 1 | ERP1 | Stilt | 2 | 0.2 | 0.2 | 4 | 2.1 | 23 | 13.5 | 38 | 30.5 | 46.3 |
| 2 | 2 | 1 | ERP1 | Stilt | 3 | 0.3 | 0.3 | 5 | 2.65 | 25 | 15 | 40 | 32.5 | 50.45 |
| 2 | 2 | 1 | ERP1 | Stilt | 4 | 0.1 | 0.1 | 7 | 3.55 | 20 | 13.5 | 35 | 27.5 | 44.65 |
| 2 | 2 | 1 | ERC | Rye | 1 | 0.5 | 0.5 | 6 | 3.25 | 12 | 9 | 32 | 22 | 34.75 |
| 2 | 2 | 1 | ERC | Rye | 2 | 0.8 | 0.8 | 7 | 3.9 | 14 | 10.5 | 33 | 23.5 | 38.7 |
| 2 | 2 | 1 | ERC | Rye | 3 | 0.6 | 0.6 | 9 | 4.8 | 17 | 13 | 35 | 26 | 44.4 |
| 2 | 2 | 1 | ERC | Rye | 4 | 0.3 | 0.3 | 9.5 | 4.9 | 15 | 12.25 | 35 | 25 | 42.45 |
| 2 | 2 | 1 | ERC | Stilt | 1 | 2 | 2 | 23 | 12.5 | 45 | 34 | 75 | 60 | 108.5 |
| 2 | 2 | 1 | ERC | Stilt | 2 | 1 | 1 | 21 | 11 | 44 | 32.5 | 74 | 59 | 103.5 |
| 2 | 2 | 1 | ERC | Stilt | 3 | 1 | 1 | 22 | 11.5 | 38 | 30 | 70 | 54 | 96.5 |
| 2 | 2 | 1 | ERC | Stilt | 4 | 2 | 2 | 26 | 14 | 40 | 33 | 72 | 56 | 105 |
| 2 | 2 | 2 | ERP1 | Rye | 1 | 3 | 3 | 24 | 13.5 | 53 | 38.5 | 80 | 66.5 | 121.5 |
| 2 | 2 | 2 | ERP1 | Rye | 2 | 4 | 4 | 33 | 18.5 | 65 | 49 | 100 | 82.5 | 154 |
| 2 | 2 | 2 | ERP1 | Rye | 3 | 5 | 5 | 21 | 13 | 45 | 33 | 80 | 62.5 | 113.5 |
| 2 | 2 | 2 | ERP1 | Rye | 4 | 3 | 3 | 29 | 16 | 63 | 46 | 93 | 78 | 143 |
| 2 | 2 | 2 | ERP1 | Stilt | 1 | 0.1 | 0.1 | 5 | 2.55 | 15 | 10 | 38 | 26.5 | 39.15 |
| 2 | 2 | 2 | ERP1 | Stilt | 2 | 0.3 | 0.3 | 6 | 3.15 | 18 | 12 | 40 | 29 | 44.45 |
| 2 | 2 | 2 | ERP1 | Stilt | 3 | 0.5 | 0.5 | 7 | 3.75 | 12 | 9.5 | 35 | 23.5 | 37.25 |
| 2 | 2 | 2 | ERP1 | Stilt | 4 | 0.4 | 0.4 | 6 | 3.2 | 10 | 8 | 33 | 21.5 | 33.1 |
| 2 | 2 | 2 | ERC | Rye | 1 | 0.5 | 0.5 | 9 | 4.75 | 17 | 13 | 33 | 25 | 43.25 |
| 2 | 2 | 2 | ERC | Rye | 2 | 0.7 | 0.7 | 8 | 4.35 | 18 | 13 | 36 | 27 | 45.05 |
| 2 | 2 | 2 | ERC | Rye | 3 | 0.5 | 0.5 | 9 | 4.75 | 16 | 12.5 | 33 | 24.5 | 42.25 |
| 2 | 2 | 2 | ERC | Rye | 4 | 0.7 | 0.7 | 8 | 4.35 | 19 | 13.5 | 32 | 25.5 | 44.05 |
| 2 | 2 | 2 | ERC | Stilt | 1 | 2 | 2 | 21 | 11.5 | 43 | 32 | 55 | 49 | 94.5 |
| 2 | 2 | 2 | ERC | Stilt | 2 | 1 | 1 | 19 | 10 | 38 | 28.5 | 50 | 44 | 83.5 |
| 2 | 2 | 2 | ERC | Stilt | 3 | 2 | 2 | 20 | 11 | 41 | 30.5 | 57 | 49 | 92.5 |
| 2 | 2 | 2 | ERC | Stilt | 4 | 2.8 | 2.8 | 12.5 | 7.65 | 30.5 | 21.5 | 42 | 36.25 | 68.2 |
| 2 | 2 | 3 | ERP1 | Rye | 1 | 4 | 4 | 35 | 19.5 | 56 | 45.5 | 81 | 68.5 | 137.5 |
| 2 | 2 | 3 | ERP1 | Rye | 2 | 6 | 6 | 38 | 22 | 58 | 48 | 85 | 71.5 | 147.5 |
| 2 | 2 | 3 | ERP1 | Rye | 3 | 6 | 6 | 37 | 21.5 | 55 | 46 | 83 | 69 | 142.5 |
| 2 | 2 | 3 | ERP1 | Rye | 4 | 4 | 4 | 20 | 12 | 40 | 30 | 76 | 58 | 104 |
| 2 | 2 | 3 | ERP1 | Stilt | 1 | 0.2 | 0.2 | 4 | 2.1 | 19.2 | 11.6 | 40 | 29.6 | 43.5 |
| 2 | 2 | 3 | ERP1 | Stilt | 2 | 0.3 | 0.3 | 5 | 2.65 | 19 | 12 | 38 | 28.5 | 43.45 |
| 2 | 2 | 3 | ERP1 | Stilt | 3 | 0.5 | 0.5 | 6 | 3.25 | 17 | 11.5 | 35 | 26 | 41.25 |
| 2 | 2 | 3 | ERP1 | Stilt | 4 | 0.3 | 0.3 | 5 | 2.65 | 15 | 10 | 35 | 25 | 37.95 |
| 2 | 2 | 3 | ERC | Rye | 1 | 0.7 | 0.7 | 11 | 5.85 | 20 | 15.5 | 34 | 27 | 49.05 |
| 2 | 2 | 3 | ERC | Rye | 2 | 0.8 | 0.8 | 9 | 4.9 | 15 | 12 | 28 | 21.5 | 39.2 |
| 2 | 2 | 3 | ERC | Rye | 3 | 0.3 | 0.3 | 12 | 6.15 | 21 | 16.5 | 35 | 28 | 50.95 |
| 2 | 2 | 3 | ERC | Rye | 4 | 0.3 | 0.3 | 10 | 5.15 | 18 | 14 | 34 | 26 | 45.45 |
| 2 | 2 | 3 | ERC | Stilt | 1 | 3 | 3 | 26 | 14.5 | 47 | 36.5 | 79 | 63 | 117 |
| 2 | 2 | 3 | ERC | Stilt | 2 | 5 | 5 | 24 | 14.5 | 41 | 32.5 | 83 | 62 | 114 |
| 2 | 2 | 3 | ERC | Stilt | 3 | 3 | 3 | 22 | 12.5 | 38 | 30 | 69 | 53.5 | 99 |
| 2 | 2 | 3 | ERC | Stilt | 4 | 2 | 2 | 20 | 11 | 35 | 27.5 | 70 | 52.5 | 93 |
| 2 | 2 | 4 | ERP1 | Rye | 1 | 4 | 4 | 37 | 20.5 | 50 | 43.5 | 78 | 64 | 132 |
| 2 | 2 | 4 | ERP1 | Rye | 2 | 6 | 6 | 39 | 22.5 | 58 | 48.5 | 73 | 65.5 | 142.5 |
| 2 | 2 | 4 | ERP1 | Rye | 3 | 5 | 5 | 36 | 20.5 | 52 | 44 | 71 | 61.5 | 131 |
| 2 | 2 | 4 | ERP1 | Rye | 4 | 4 | 4 | 35 | 19.5 | 50 | 42.5 | 70 | 60 | 126 |
| 2 | 2 | 4 | ERP1 | Stilt | 1 | 0.2 | 0.2 | 4 | 2.1 | 36 | 20 | 52 | 44 | 66.3 |
| 2 | 2 | 4 | ERP1 | Stilt | 2 | 0.3 | 0.3 | 5 | 2.65 | 38 | 21.5 | 53 | 45.5 | 69.95 |
| 2 | 2 | 4 | ERP1 | Stilt | 3 | 0.5 | 0.5 | 6.4 | 3.45 | 42 | 24.2 | 58 | 50 | 78.15 |
| 2 | 2 | 4 | ERP1 | Stilt | 4 | 0.3 | 0.3 | 5 | 2.65 | 38 | 21.5 | 52 | 45 | 69.45 |
| 2 | 2 | 4 | ERC | Rye | 1 | 0.2 | 0.2 | 9 | 4.6 | 16 | 12.5 | 35 | 25.5 | 42.8 |
| 2 | 2 | 4 | ERC | Rye | 2 | 0.3 | 0.3 | 8 | 4.15 | 15 | 11.5 | 34 | 24.5 | 40.45 |
| 2 | 2 | 4 | ERC | Rye | 3 | 0.2 | 0.2 | 8 | 4.1 | 15 | 11.5 | 35 | 25 | 40.8 |
| 2 | 2 | 4 | ERC | Rye | 4 | 0.1 | 0.1 | 7 | 3.55 | 10 | 8.5 | 30 | 20 | 32.15 |
| 2 | 2 | 4 | ERC | Stilt | 1 | 3 | 3 | 32 | 17.5 | 46 | 39 | 60 | 53 | 112.5 |
| 2 | 2 | 4 | ERC | Stilt | 2 | 4 | 4 | 30 | 17 | 48 | 39 | 63 | 55.5 | 115.5 |
| 2 | 2 | 4 | ERC | Stilt | 3 | 3 | 3 | 28 | 15.5 | 42 | 35 | 55 | 48.5 | 102 |
| 2 | 2 | 4 | ERC | Stilt | 4 | 5 | 5 | 24 | 14.5 | 38 | 31 | 53 | 45.5 | 96 |
